# Long-term hematopoietic stem cells as a parasite niche during treatment failure in visceral leishmaniasis

**DOI:** 10.1038/s42003-022-03591-7

**Published:** 2022-06-25

**Authors:** Laura Dirkx, Sarah Hendrickx, Margot Merlot, Dimitri Bulté, Marick Starick, Jessy Elst, André Bafica, Didier G. Ebo, Louis Maes, Johan Van Weyenbergh, Guy Caljon

**Affiliations:** 1 Laboratory of Microbiology, Parasitology, and Hygiene (LMPH), Infla-Med Centre of Excellence, University of Antwerp, Antwerp, Belgium; 2 Clinical and Epidemiological Virology, Department of Microbiology, Immunology, and Transplantation, Rega Institute of Medical Research, KU Leuven, Leuven, Belgium; 3 Laboratory of Immunobiology, Department of Microbiology, Immunology and Parasitology Federal University of Santa Catarina, Florianopolis, Brazil; 4 Department of Immunology—Allergology—Rheumatology, Faculty of Medicine and Health Science and the Infla-Med Centre of Excellence, University of Antwerp, Antwerp University Hospital, Antwerp, Belgium

**Keywords:** Parasitology, Immunology

## Abstract

Given the discontinuation of various first-line drugs for visceral leishmaniasis (VL), large-scale in vivo drug screening, establishment of a relapse model in rodents, immunophenotyping, and transcriptomics were combined to study persistent infections and therapeutic failure. Double bioluminescent/fluorescent *Leishmania infantum* and *L. donovani* reporter lines enabled the identification of long-term hematopoietic stem cells (LT-HSC) as a niche in the bone marrow with remarkably high parasite burdens, a feature confirmed for human hematopoietic stem cells (hHSPC). LT-HSC are more tolerant to antileishmanial drug action and serve as source of relapse. A unique transcriptional ’StemLeish’ signature in these cells was defined by upregulated TNF/NF-κB and RGS1/TGF-β/SMAD/SKIL signaling, and a downregulated oxidative burst. Cross-species analyses demonstrated significant overlap with human VL and HIV co-infected blood transcriptomes. In summary, the identification of LT-HSC as a drug- and oxidative stress-resistant niche, undergoing a conserved transcriptional reprogramming underlying *Leishmania* persistence and treatment failure, may open therapeutic avenues for leishmaniasis.

## Abstract (web summary)

Long-term hematopoietic stem cells may act as protective niches for the *Leishmania* parasite, potentially contributing to treatment failure in cases of visceral leishmaniasis.

## Introduction

Visceral leishmaniasis (VL) is a lethal neglected tropical disease caused by the obligate intracellular protozoan *Leishmania*^1,2^ and is transmitted through the bites of infected female phlebotomine sand flies^3,4^. In the vertebrate host, parasites propagate as amastigotes in monocyte-derived cells of the liver, spleen, and bone marrow (BM), and eventually may cause life-threatening complications^5–7^.

Successful curative treatment of VL is notoriously challenging. A particularly alarming situation is the increasing number of VL infections that are inadequately responding to most of the known antileishmanial drugs^8^. To date, antimony (Sb) and miltefosine (MIL) monotherapy against VL have been discontinued in the Indian subcontinent due to an increase in the number of treatment failures and/or relapses, with a staggering paromomycin (PMM) relapse rate of 17% and MIL relapse rates up to 20% within 12 months after treatment^9,10^. AmBisome (liposomal amphotericin B) is currently used in the VL elimination campaign in the Indian subcontinent, despite the logistic requirement of a cold-chain and its possible association with higher post-kala-azar dermal leishmaniasis (PKDL) incidence^11^. In general it can be concluded that therapeutic failure occurs frequently and treatment options become critically compromised^12^. Associations are often sought with drug resistance, although microorganisms can also be impervious to drugs without selection of genetic mutations. The formation of viable non-replicating cells, also known as persister-like cells, is common among bacteria and has also been described for *Plasmodium falciparum* and *P. vivax*, *Toxoplasma gondii*, *Trypanosoma cruzi, Leishmania* spp. and *Mycobacterium tuberculosis*^13^. Persistent infections can occur in different tissues and cells throughout the host, such as hepatocytes (*P. vivax*), skeletal muscle and neurons (*T. gondii*), adipose tissue (*T. cruzi*) and the BM (*M. tuberculosis*)^14–18^. Some of these niches give protection against active immunity and drug action^13^. Within the BM, VL parasites are renowned to elicit emergency hematopoiesis with consequent exhaustion of hematopoietic stem cells (HSC)^19–21^ and induction of leucopenia, neutropenia, thrombocytopenia, and anemia^22^.

Own historical drug evaluation data triggered the hypothesis that treatment is generally less effective in the BM hence representing a reservoir site from where relapse can occur. To date, *Leishmania* spp. have been extensively reported to reside primarily within macrophages and dendritic cells^23^ and case studies document infested macrophages in BM aspirates^24–26^. However, the exact pre- and post-treatment tropism in the BM has not yet been exhaustively described. In this study, LT-HSC were found to become readily infected, which impedes efficacy of a range of antileishmanial drugs, unrelated to efflux pumps. This indicates that infection of LT-HSC may not only allow escape from the host immune response^27^ but also from antileishmanial drug action. Our cross-species omics analysis revealed a unique but evolutionary conserved RGS1/TGF-β/SKIL signaling-driven StemLeish gene signature. This unique StemLeish gene signature corresponded to a functional *RGS1*^*high*^*TNFAIP3*^*high*^*NOS2*^*low*^ profile in *Leishmania* infected HSC, with decreased levels of reactive oxygen species (ROS) and nitric oxide (NO) that represent important effector molecules involved in intracellular pathogen killing^28,29^. In summary, this study identified a protective cellular niche of persistent *Leishmania* parasites in the BM. Given the current field situation of increasing post-treatment relapse rates, our findings warrant for advanced pharmacodynamic and drug exploration.

## Results

### Bone marrow as a niche for persistent VL parasites

**Fig. 1:**
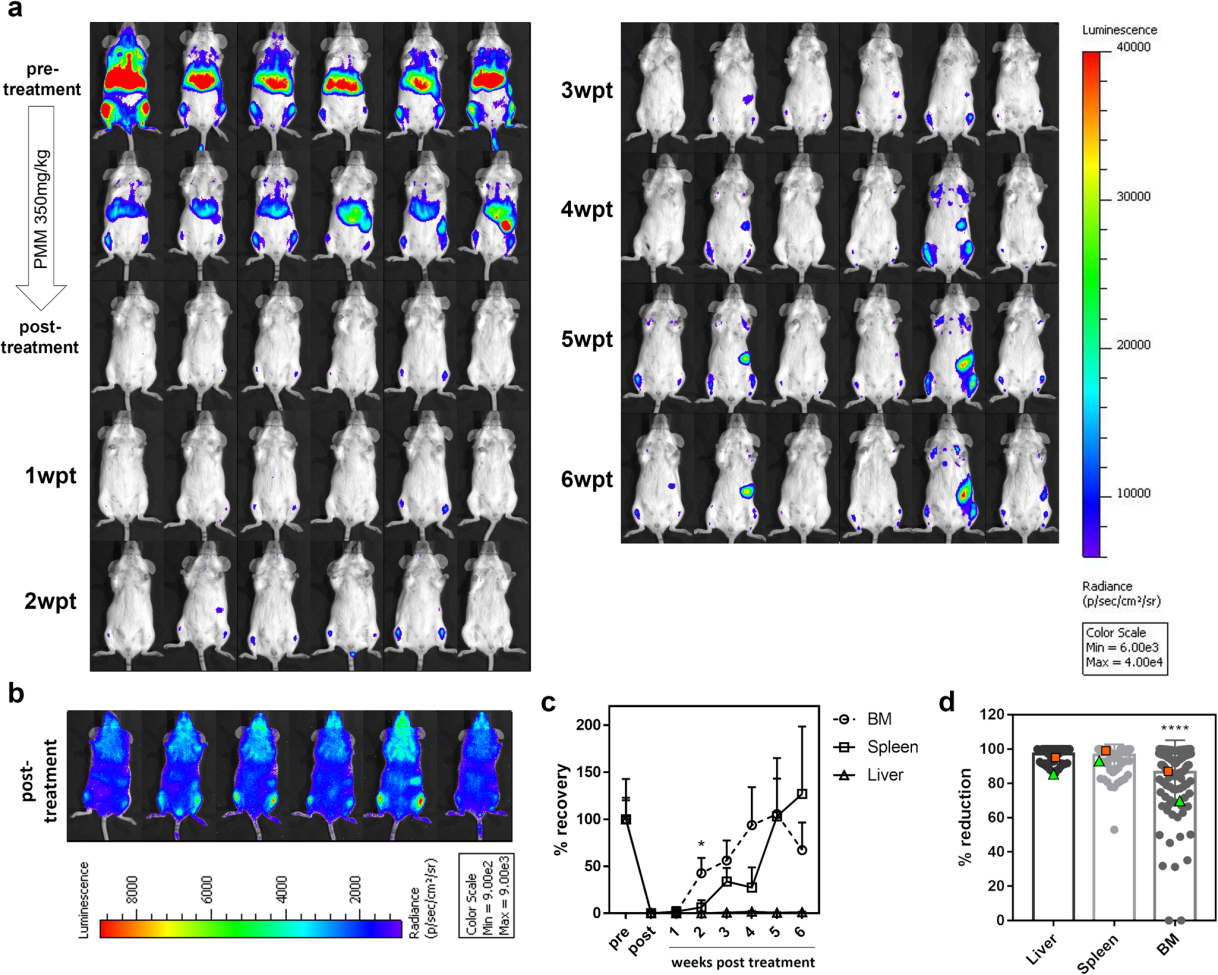
Reproducible post-treatment relapse model using sub-curative paromomycin identifies the BM as niche for treatment failure. **a** In vivo bioluminescent imaging (BLI) using an exposure time of 15 min of LEM3323 WT^*PpyRE9*^ infected BALB/c mice at 1-6 weeks post-treatment (wpt) at 350 mg/kg PMM *s.i.d.* IP for 5 days. **b** BLI (with sensitivity scale) at the end of treatment. **c** Mean relative luminescence units (RLU) values of BM and spleen during the first 6 weeks of LEM3323 WT^*PpyRE9*^ infection in BALB/c mice, where 100% is the pretreatment RLU and 0% the post-treatment RLU. Results are expressed as mean ± SEM, Mann-Whitney test (two-tailed), *p ≤ 0.05. **a**–**c** Groups consist of 3-6 BALB/c mice (three independent experiments). **d** Reduction in parasite burden after treatment of golden Syrian hamsters with a broad set (*n* = 90) of antileishmanial test compounds; treatments with >85% clearance in the liver were considered therapeutically relevant. As a reference, squares (orange) depict results with MIL, and triangles (green) represent SSG treatment. Wilcoxon matched-pairs signed rank test (two-tailed), ****p ≤ 0.0001.

By means of in vivo bioluminescence imaging (BLI), we developed a reproducible post-treatment relapse model in BALB/c mice using PMM exposure. After five consecutive days of 350 mg/kg *s.i.d*. intraperitoneal (i.p.) injections of PMM, a significant decrease of parasite burdens in the liver, spleen, and BM was obtained (Fig. 1a) indicating that PMM is able to reach these infection sites. However, low-level BM burdens above the detection threshold remained at the end of treatment as evidenced by imaging at a high sensitivity scale. In animals with the highest BM signal, bioluminescent signals also appear in the spleen (Fig. 1b). These burdens re-emerged in the absence of drug pressure indicating that BM represents a niche where parasites can survive treatment. Subsequently, parasites were able to recolonize target organs with BLI signals increasing from 2 to 3 weeks post-treatment (wpt) onwards in the spleen. Remarkably, the liver seems to be protected from recolonization (Fig. 1c). As BLI is a semi-quantitative method, RT-qPCR was performed to address the sensitivity of imaging different organs showing that burdens are relatively underestimated in the BM compared to the spleen, possibly due to the low perfusion rate or lower accessibility of the BM for the D-luciferin substrate^30^ (Supplementary Fig. 1b). Besides the BALB/c model, the golden Syrian hamster model of symptomatic progressive VL was used as it is considered more representative for human VL and appropriate for studying drug efficacy and relapse. Own historical laboratory data in the early curative VL hamster model using diverse experimental compounds and reference drugs revealed that the BM was indeed the most difficult to clear. In extreme cases, no drug activity was recorded in the BM at all (Fig. 1d and Supplementary Data 1). For instance, the 8-aminoquinoline analogs Sitamaquine (compound 10) and Tafenoquine (compound 86) were found to be inactive in the BM at effective concentrations in the liver (Supplementary Data 1).

### Identification of specific BM cell niches for viable *Leishmania* parasites during infection and upon relapse

**Fig. 2:**
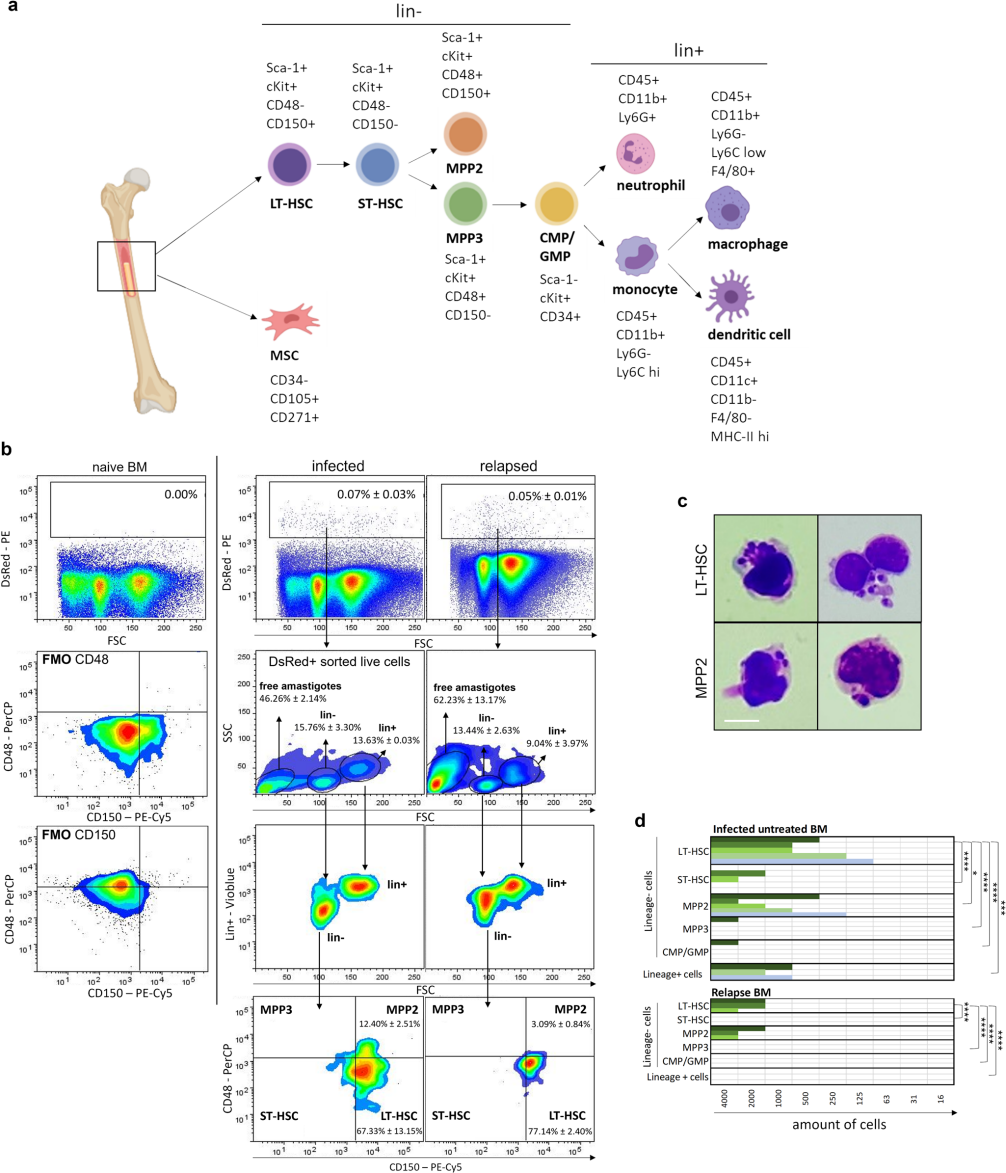
Identification of LT-HSC and MPP2 as niche for viable parasites during infection and following treatment failure. **a** Specific markers for BM cell subsets: long-term hematopoietic stem cell (LT-HSC), short-term HSC (ST-HSC), multipotent progenitor (MPP), common myeloid progenitor (CMP), granulocyte-monocyte progenitor (GMP), mesenchymal stem cells (MSC). **b** Cell sorting of DsRed^+^ BM cells of LEM3323 WT^*PpyRE9/DsRed*^ infected BALB/c mice to enrich the infected sample, followed by re-measuring with markers as described in (**a**) and gated on LSK cells, i.e., Lin^−^, Sca-1^+^ and cKit^+^ cells. Fluorescence minus one (FMO) controls are displayed to confirm the gates. Representative plots and frequencies of DsRed^+^ cells (mean ± SD) are shown of two independent experiments per condition (*n* = 4), performed at 4 wpi (infected) and 5 wpt (relapsed). **c** Giemsa-stained LT-HSC and MPP2 cells sorted from LEM3323 WT^*PpyRE9/DsRed*^ infected BALB/c mice (6wpi). Scale bar = 10 µm. **d** Results of promastigote back-transformation assays after FACS of an indicated number of cells (LT-HSC, ST-HSC, MPP2, MPP3, CMP/GMP, and Lin^+^ cells) from infected untreated mice (top panel, pooled results of 4, 5 and 6 wpi) and relapsed mice after PMM treatment (bottom panel, pooled results of 3, 4 and 5 wpt). Colored bars represent independent experiments (3 ≤ *n* ≤ 5) and positive back-transformation. Statistical significance levels using titration endpoints are indicated (2-way ANOVA, multiple comparisons). *p < 0.05, ***p < 0.001, ****p < 0.0001. The diagram in (**a**) was generated using Biorender.com.

Progenitor and stem cells only represent 0.01% of the total BM, hence requiring enrichment for analysis in the complex pool of BM cellular constituents^31^. To identify the specific BM cell subsets in which the parasites reside in vivo, a two-step enrichment was performed combined with a staining for specific markers (Fig. 2a). Cells harboring a DsRed^+^ fluorescent signal from *Leishmania* strain were flow sorted, stained and analyzed excluding lineage positive cells (Ter-119, Ly-6C/G, CD11b, TCR-β, B220, and CD335) as second enrichment step. Here, *Leishmania* sensitive BM cells were identified, i.e., long-term hematopoietic stem cells (LT-HSC; Lin^−^ Sca1^+^ cKit^+^ CD48^−^ CD150^+^) and to a lesser extent multipotent progenitors 2 (MPP2; Lin^−^ Sca1^+^ cKit^+^ CD48^+^ CD150^+^). As in vivo infection in the BM only comprises 0.07 ± 0.03% of total cells, detection of infected stem cells is scarce and easily overlooked (Fig. 2b). In Fig. 2b all DsRed^+^ cells are shown to illustrate the presence of events that correspond to extracellular amastigotes (46.26% ± 2.14% in infected mice, 62.23% ± 13.17% in relapsed mice), lineage positive cells (13.63% ± 0.03% in infected mice, 9.04% ± 3.97% in relapsed mice) and lineage negative cells (15.76% ± 3.30% in infected mice, 13.44% ± 2.63% in relapsed mice) that mainly consist of LT-HSC (67.33% ± 13.15% in infected mice, 77.14% ± 2.40% in relapsed mice). Although the occurrence of extracellular amastigotes could be the consequence of the sorting and centrifugation procedure, this has also been described in BM smears of patients together with the presence in lineage positive cells^32,33^. The release of extracellular amastigotes that survived drug treatment could next initiate reinfection of BM cells. The percentage in vivo LT-HSC infection was calculated to be 20.04% ± 7.67%, in a comparable range to what is observed in ex vivo infections of LT-HSC (*vide infra*). For relapsed mice after PMM treatment, in vivo infection in the BM is 0.05% ± 0.01% of total cells, and the percentage LT-HSC infection is 6.67% ± 0.97% (Fig. 2b).

Giemsa staining also revealed that LT-HSC, with confirmed post-sort purity of 95.3% (Supplementary Fig. 2a), harbored more parasites than MPP2 (Fig. 2c). To additionally confirm amastigote viability in the various BM cells of infected and relapsed mice, a promastigote back-transformation assay was performed as most reliable indicator of viability^34^. A formal link and the relative contribution of LT-HSC during infection and relapse in vivo was thus explored using cell sorting of lineage positive cells and various stem cell types from the BM of infected/treated mice. After PMM treatment, LT-HSC and MPP2 were still found to harbor viable parasites, whereas no viable parasites could be recovered from sorted lineage positive cells (Fig. 2d). Similar results were obtained at 3, 4, and 5 weeks post treatment and coincided with the gradual detection of relapse by BLI. Promastigote back-transformation, which provides more accurate data than the dsRed^+^ signals on parasite viability, unequivocally demonstrated that LT-HSC reproducibly harbored high numbers of viable parasites post-treatment (Fig. 2d).

### LT-HSC are exceptionally susceptible host cells to VL infection

**Fig. 3:**
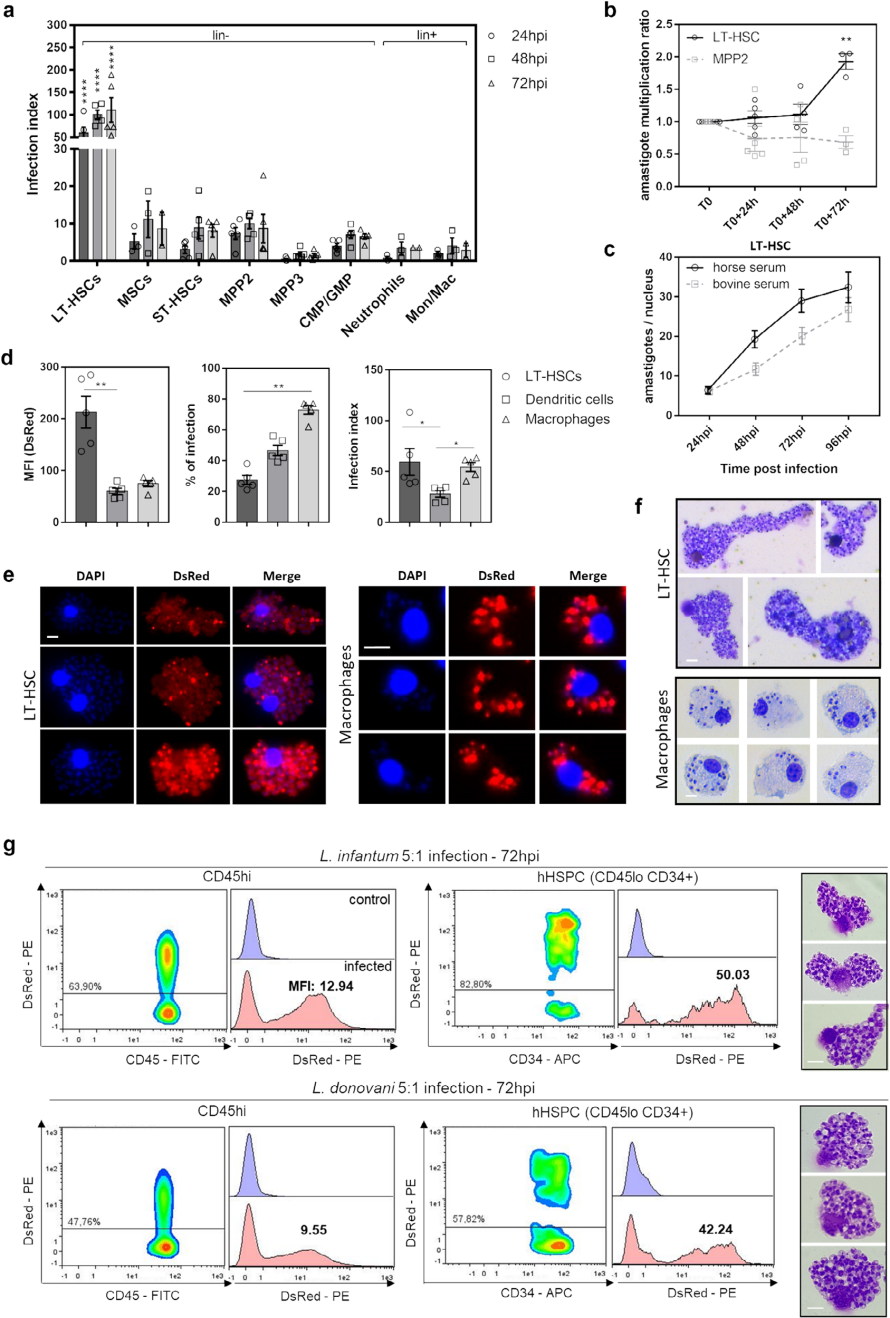
Ex vivo LT-HSC are most susceptible to VL infection in vitro. **a** Ex vivo LEM3323 WT^*PpyRE9/DsRed*^ infection of lineage depleted BM collected from BALB/c mice: infection index representing DsRed MFI × % of infection. Statistical comparisons were made between LT-HSC and all other groups, for each time point. **b** Evolution over time of the DsRed MFI indicating intracellular parasite expansion (amastigote multiplication ratio) in two BM cell subsets: LT-HSC and MPP2. **c** Additional confirmation of amastigote multiplication using horse serum versus bovine serum on LEM3323 WT^*PpyRE9/DsRed*^ infected sorted LT-HSC; amastigotes per nucleus microscopically counted after Giemsa staining (*n* = 30 cells). **d** Comparison of MFI, % infection, and infection index between BM-derived dendritic cells and macrophages and LT-HSC. **e** Immunofluorescence microphotographs of LEM3323 WT^*PpyRE9/DsRed*^ infected (120 h) BM-derived macrophages and sorted LT-HSC with nuclei/kinetoplasts stained with DAPI (blue) and DsRed signal from LEM3323 WT^*PpyRE9/DsRed*^ (red), scale bar = 10 µm. **f** Giemsa staining of infected BM-derived macrophages and sorted LT-HSC, scale bar = 10 µm. **g** DsRed plots of CD45^hi^ and hHSPC (CD45^lo^ CD34^+^) BM subsets and histogram of representative results for three human bone marrow aspirates, ex vivo infected for 72 h using either *L. infantum* (LEM3323 WT^*PpyRE9/DsRed*^) or *L. donovani* (L82 WT^*PpyRE9/DsRed*^) promastigotes. % of infection and median fluorescence intensity (MFI) is provided in the individual plots. Giemsa confirmation of infection of hHSPCs, at 72 hpi, with both *L. infantum* and *L. donovani* promastigotes, is shown on the right. Scale bar = 10 µm. **a**–**g** Days/hours post-infection (dpi/hpi), median fluorescence intensity (MFI), mesenchymal stem cell (MSC), long-term hematopoietic stem cell (LT-HSC), short-term hematopoietic stem cell (ST-HSC), multipotent progenitor (MPP), common myeloid progenitor (CMP), granulocyte-monocyte progenitor (GMP), monocyte/macrophage (Mon/Mac), human hematopoietic stem cell (hHSPC). Results are shown as mean ± SEM and are based on at least three independent experiments (3 ≤ *n* ≤ 5). Statistical significance was found with two-tailed tests, i.e., 2-way ANOVA in (**a**), multiple *t* test illustrating a difference between LT-HSC and MPP2 in (**b**), and Kruskal–Wallis in (**d**). *p < 0.05, **p < 0.01, ****p < 0.0001.

To further investigate the intrinsic susceptibility of BM cell subsets to *Leishmania* infection, stem cells were isolated by negative immunomagnetic selection, followed by ex vivo infection with *Leishmania*. Lineage negative BM cells were co-cultured with metacyclic *L. infantum* (LEM3323 WT^*PpyRE9/DsRed*^) promastigotes for 24, 48, and 72 h, followed by flow cytometry analysis. Compared to the various BM cell subsets, LT-HSC were more readily infected with *Leishmania*, both in terms of proportion of infected cells (% of infection) and the number of amastigotes per cell based on the DsRed median fluorescence intensity (MFI) (Fig. 3a and Supplementary Fig. 3a). Interestingly, the MFI of infected LT-HSC increased over time compared to *T*_0_ (24 hpi) implying that these cells are highly permissive for amastigote multiplication, in contrast to the stagnating parasite burdens in MPP2 cells (Fig. 3b). This intracellular amastigote multiplication was confirmed by Giemsa staining in a comparative experiment using horse and bovine serum (Fig. 3c), the former known to remove extracellular promastigotes as potentially confounding factor to determine intracellular multiplication^35^. Compared to BM-derived macrophages and dendritic cells as established host cells for *Leishmania*, LT-HSC infection rates were lower (±20%, comparable to in vivo infection) whereas the MFI was substantially higher, hence representative of considerably higher intracellular burdens (Fig. 3d). Strikingly high amastigote burdens were further revealed in sorted LT-HSC using immunofluorescence and Giemsa analyses (Fig. 3e, f). The DsRed signal was more variable amongst the amastigotes in LT-HSC which may indicate the occurrence of quiescence in a subset of the parasites. The high susceptibility of LT-HSC was confirmed using the *L. donovani* L82 WT^*PpyRe9/DsRed*^ strain (Supplementary Fig. 4a–d) and using amastigotes of both *L. infantum* LEM3323 WT^*PpyRE9/DsRed*^ and *L. donovani* L82 WT^*PpyRE9/DsRed*^ (Supplementary Fig. 3b). Cross-species confirmation using human BM aspirates from three different healthy donors confirmed heavy VL infection of hHSPC (CD45^lo^ CD34^+^) after in vitro infection comparable to the observations in mouse LT-HSC (Fig. 3g).

### LT-HSC represent an oxidative stress- and drug-resistant sanctuary for viscerotropic *Leishmania spp*

**Fig. 4:**
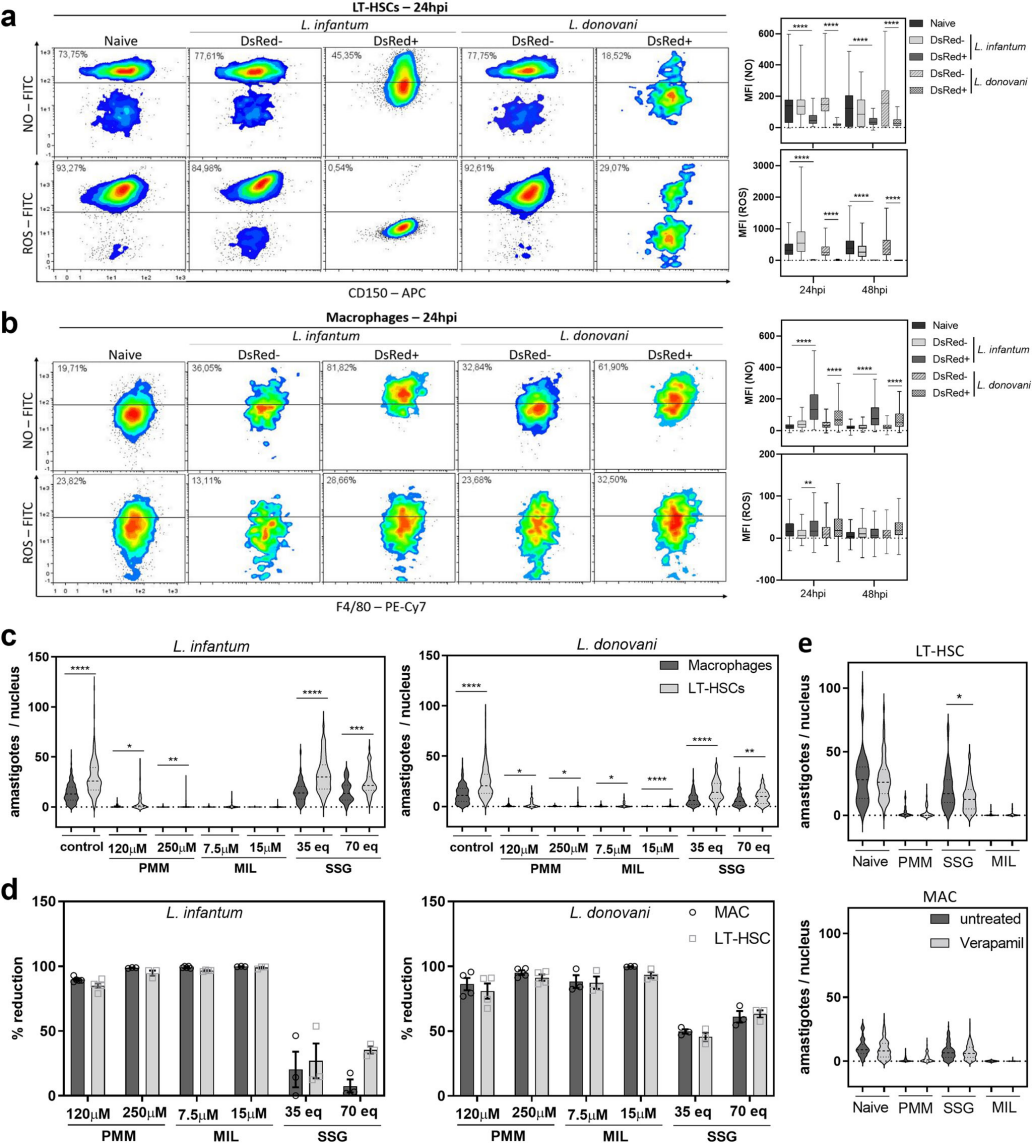
LT-HSC represent an oxidative stress- and drug-resistant sanctuary for viscerotropic *Leishmania* spp. **a**, **b** ROS and NO levels, determined using CM-H_2_DCFDA and DAF-FM diacetate, are decreased in ex vivo infected stem cells. Density plots of LT-HSC (**a**) and macrophages (**b**) at 24 h post-infection (hpi) with fluorescent signal of NO (top rows) and ROS (bottom rows). Graph represents the MFI of these plots at 24 and 48 h post-infection. Representative dot plots with corresponding graphs of three independent experiments with similar results are shown. Multiple *t* tests (two-tailed) after outlier analysis (ROUT, *Q* = 1%), boxplots represent minimum, maximum, median and 75% and 25% percentiles, 155 ≤ *n* ≤ 16250, **p < 0.01, ****p < 0.0001. **c** Sorted LT-HSC infected with *L. donovani* L82 WT^*PpyRE9/DsRed*^ in a 5:1 ratio and treated for 120 h. Representative plots of two independent experiments for PMM (120 and 250 µM), MIL (7.5 and 15 µM), and SSG (35 eq. Sb and 70 eq. Sb) are shown. Confirmatory results with PMM, MIL, and SSG on *L. infantum* LEM3323 (with inherent Sb resistance) in two independent experiments. Results are expressed as mean ± SEM. Mann-Whitney test, 45 ≤ *n* ≤ 250, *p < 0.05, **p < 0.01, ****p < 0.0001. **d** Percentage of reduction compared to untreated controls is shown, calculated from the data shown in (**c**). **e** Effect of 120 h co-incubation of verapamil (8 µM) with PMM (120 µM), MIL (7.5 µM), and SSG (35 eq. Sb) on *L. infantum* LEM3323 WT^*PpyRE9/DsRed*^ (with inherent Sb resistance) infected (5:1) sorted LT-HSC and BM-derived macrophages. Results are based on three independent experiments. Mann–Whitney test, 60 ≤ *n* ≤ 100, *p < 0.05.

Given the importance of reactive nitrogen and oxygen radicals as antiparasitic response, their production in LT-HSC was examined using flow cytometry including all necessary fluorescence minus one (FMO) controls to assure specificity and exclude autofluorescence. From 24 h of ex vivo infection onwards, intracellular NO and ROS levels notably declined (*p* < 0.0001) in DsRed^+^ LT-HSC compared to naive cells or DsRed^−^ cells within the same well (Fig. 4a). In contrast, NO and ROS levels were elevated or stable in infected macrophages (Fig. 4b).

To further document LT-HSCs as a privileged cellular niche for *Leishmania*, their sensitivity to PMM, MIL and SSG was tested. Sorted LT-HSC and BM-derived macrophages were infected and drug-exposed for 120 h at two concentrations selected based on previous research^36^. In comparison to macrophages, *L. infantum* and *L. donovani* parasites remain more abundant in LT-HSC after treatment with PMM, MIL, and SSG (Fig. 4c). As the percentage of reduction remains similar between the two host cell types, it is particularly the extreme amastigote burden that impedes parasite elimination from LT-HSCs rather than an intrinsic cellular drug resistance phenotype (Fig. 4d). High-level expression of efflux pumps on stem cells could potentially contribute to the enhanced tolerance to drugs^37^. The role of efflux pumps in LT-HSC was assessed by co-incubation with the efflux pump inhibitor verapamil, as previously described^38^. Verapamil inhibits efflux mediated by both multi-drug resistance protein (MDR) and multi-drug resistance associated protein (MRP), which are often implicated in resistance. Co-administration of verapamil (8 µM) (Fig. 4e) did not enhance susceptibility of LT-HSC to most reference drugs except for SSG, a known substrate of efflux transport^39^. Through the use of verapamil, SSG efflux was shown to be more prominent in LT-HSC than in macrophages confirming previous reports of higher efflux pump expression levels in progenitors^40^. Nevertheless, parasite burden on a per-cell basis was significantly higher in LT-HSC *vs*. macrophages for all three drugs, confirming the in vivo relapse data and implicating LT-HSC as highly permissive host cells that support post-treatment survival of the intracellular parasites.

### *Leishmania infantum* infection of LT-HSC triggers a unique StemLeish transcriptional profile characterized by TNF/NF-κB and RGS1/TGF-β/SMAD/SKIL signaling

**Fig. 5:**
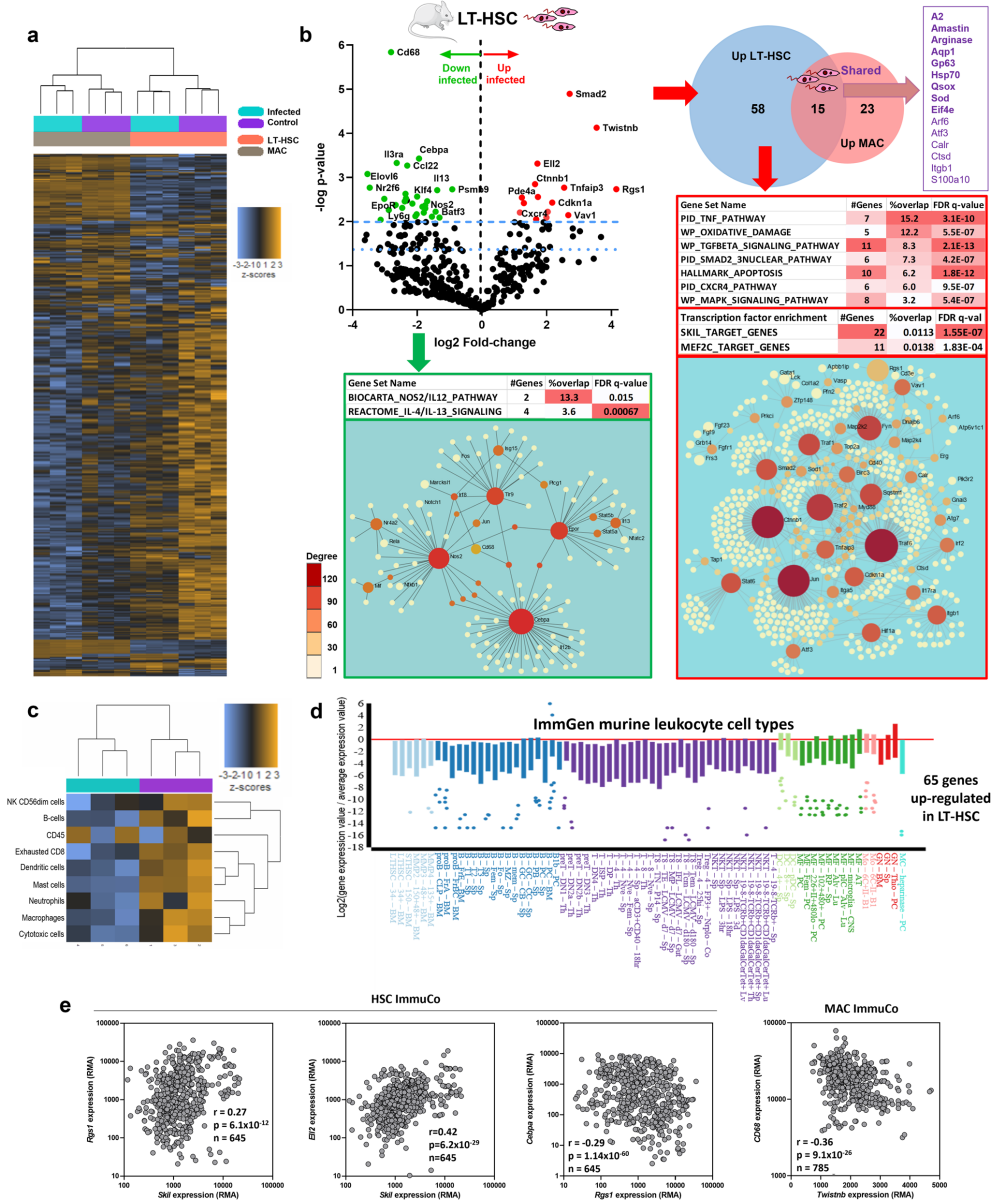
*L. infantum* infection of LT-HSC triggers a unique StemLeish transcriptional profile characterized by RGS1/TGF-β/SMAD/SKIL signaling. **a** Heatmap of 736 host and pathogen transcripts above the detection threshold and quantified by nCounter digital transcriptomics in control *vs*. *L. infantum*-infected LT-HSC (Lin^−^cKit^+^Sca1^+^CD48^−^CD150^+^) and macrophages, purified as detailed in “Methods”. **b** Volcano plot (upper left panel) and systems biology analysis of differentially expressed genes (DEG, upper left) in LT-HSC. Venn Diagram of overlapping DEG in LT-HSC *vs*. macrophages (upper right panel). Enrichment of pathways, transcription factor motifs (MSigDb), and network analysis (NetworkAnalyst 3.0) in upregulated (lower right panel) and downregulated (lower left panel) genes in LT-HSC. Genes are colored according to node degree (number of connections)^87^. **c** In silico cell type deconvolution (nSolver) of *Leishmania*-infected *vs*. uninfected LT-HSC demonstrates down-regulation of major leukocyte subsets, but not total leukocytes (*pan*-leukocytes quantified as CD45^+^). **d** Quantification of the 65-gene StemLeish upregulated subset across all leukocyte subsets (analyzed by single-cell RNAseq) in the ImmGen data set demonstrates significant downregulation in all subsets except thioglycolate-induced inflammatory neutrophils. **e** Validation of the StemLeish signature (*Rgs1*/*Skil*/*Ell2*/*Cebpa*/*Twistnb*) in large independent murine LT-HSC and macrophage datasets (ImmuCo; *n* = 645 and *n* = 785 mice respectively), analyzed using Spearman’s correlation between up- *vs*. downregulated StemLeish genes. Data are shown as RMA (Robust Microarray Average), with a conservative detection limit at RMA > 100. The highly significant positive correlations (after FDR correction) between genes upregulated in StemLeish (*Rgs1/Skil/Ell2*, two left panels) confirm that these genes are co-regulated in a quantitative manner across a large number of independent samples. A negative correlation (two right panels) was found between the upregulated *Rgs1* and *Twistnb* transcription factors, and the downregulated *Cebpa* transcription factor and *Cd68* macrophage lineage marker. Clipart used in diagram (**b**) was obtained from Servier Medical Art (https://smart.servier.com).

To unravel the molecular basis for the high susceptibility and map the comprehensive impact of infection on HSC functionality, nCounter digital transcriptomics was used on ex vivo infected LT-HSC in comparison with BM-derived macrophages, using the Mouse Myeloid Innate Immunity panel from Nanostring. As shown in a heat map of 736 genes (Fig. 5a), the overall transcriptional profile of macrophages was only modestly affected by infection (as previously demonstrated by ref. ^41^) whereas a strong effect was observed in LT-HSC. PCA analysis (Supplementary Fig. 2b) and evaluation of cell type-specific transcripts (Supplementary Fig. 2c) revealed distant profiles of the LT-HSC and macrophage samples, further supporting the very high purity of the sorted cells. A total of 101 genes were differentially expressed in LT-HSC (73 up- and 28 downregulated, Fig. 5b and Supplementary Data 2a) in contrast to 49 differentially expressed genes in macrophages (38 up- and 11 downregulated, Supplementary Data 2b). Of the 15 upregulated genes shared between LT-HSC and macrophages, 9 corresponded to *Leishmania* transcripts whereas only 6 were host genes (*Arf6*, *Atf3*, *Calr*, *Ctsd*, *Itgb1*, and *S100a10*). Downregulated genes (Fig. 5b) included notably monocyte/macrophage lineage marker *Cd68*, transcriptional regulator *Cepba*, genes involved in IL-4/IL-13 signaling, and *Nos2* (Fig. 5b), corroborating our in vitro assays (Fig. 4). None of the downregulated genes were shared between LT-HSC and macrophages, further confirming the divergent response to infection in both cell types. A systems biology analysis of LT-HSC upregulated genes revealed significant enrichment of several hallmark gene sets (Supplementary Data 2c), curated pathways (Supplementary Data 2d), and transcription factor motifs (Supplementary Data 2e). The most predominant pathways were TNF/NF-κB, TFG-β/SMAD/SKIL and MAPK signaling as well as oxidative damage/reactive nitrogen and oxygen species detoxification, and p53-mediated apoptosis (Fig. 5b, lower right panel), all of which have previously been demonstrated in either human or experimental VL^29,41–46^.

However, nCounter profiling also revealed several highly upregulated LT-HSC genes that have not yet been described in *Leishmania spp*. infections, such as *Rgs1*, *Twistnb*, *Ell2*, *Vav1*. Three orthogonal approaches argue in favor of a new HSC transcriptional program (further referred to as ’StemLeish’), rather than a *Leishmania*-triggered differentiation of LT-HSC to one or more specific leukocyte subsets. First, in silico cell type deconvolution of *Leishmania*-infected *vs*. uninfected LT-HSC demonstrates downregulation of major leukocyte subsets, but not total leukocytes (*pan*-leukocytes quantified as CD45^+^) (Fig. 5c and Supplementary Data 2f). Second, quantification of the 65-gene StemLeish upregulated subset across all currently known leukocyte subsets (including several HSC subsets) analyzed by RNAseq in the comprehensive ImmGen data set (Fig. 5d), demonstrated significant downregulation in all subsets (except thioglycolate-induced inflammatory neutrophils, for which a null result was obtained, i.e., no significant up- or downregulation). Similar results were obtained for StemLeish downregulated genes (Supplementary Fig. 5a and Supplementary Data 2g, h). Third, transcription factor enrichment demonstrated ‘classical’ pro-inflammatory NF-κB and AP-1 (Jun/Fos) transcription factors, but also Skil, Mef2c, Foxf2, Bach2 (Supplementary Data 2e) which have not been previously described in either experimental or human leishmaniasis.

We therefore further investigated the StemLeish gene signature in large publicly available transcriptomic datasets (ImmuCo) of purified mouse leukocyte subsets. In agreement with our results, *Rgs1*, *Skil*, *Ell2*, *Cebpa,* and *Twistnb* were confirmed as highly expressed in murine HSCs isolated from >600 animals (Fig. 5e). We next verified how both genes might be regulated in HSC differentiation towards myeloid, lymphoid, and erythroid lineages. *Rgs1* was found to be significantly positively correlated with *Ell2* and *Skil* in purified HSCs. In line with our nCounter digital transcriptomics data, *Rgs1* negatively correlated in purified HSCs to the master transcriptional regulator *Cebpa* (*p* = 1.4 × 10^−60^), a driver of myeloid and particularly neutrophil differentiation. In addition, *Twistnb* was also negatively correlated (*p* = 9.1 × 10^−^^26^) to *Cd68* transcript levels in macrophages. Taken together, overexpression of *Rgs1* and transcription factors *Twistnb/Vav1/Smad2/Ell2/Atf3* characterize a StemLeish transcriptional program in LT-HSC that differs from previously described transcriptional reprogramming triggered by *Leishmania spp*^21,41–43,47^.

### The StemLeish gene signature recapitulates cross-species epigenetic and transcriptional signatures of visceral leishmaniasis

**Fig. 6:**
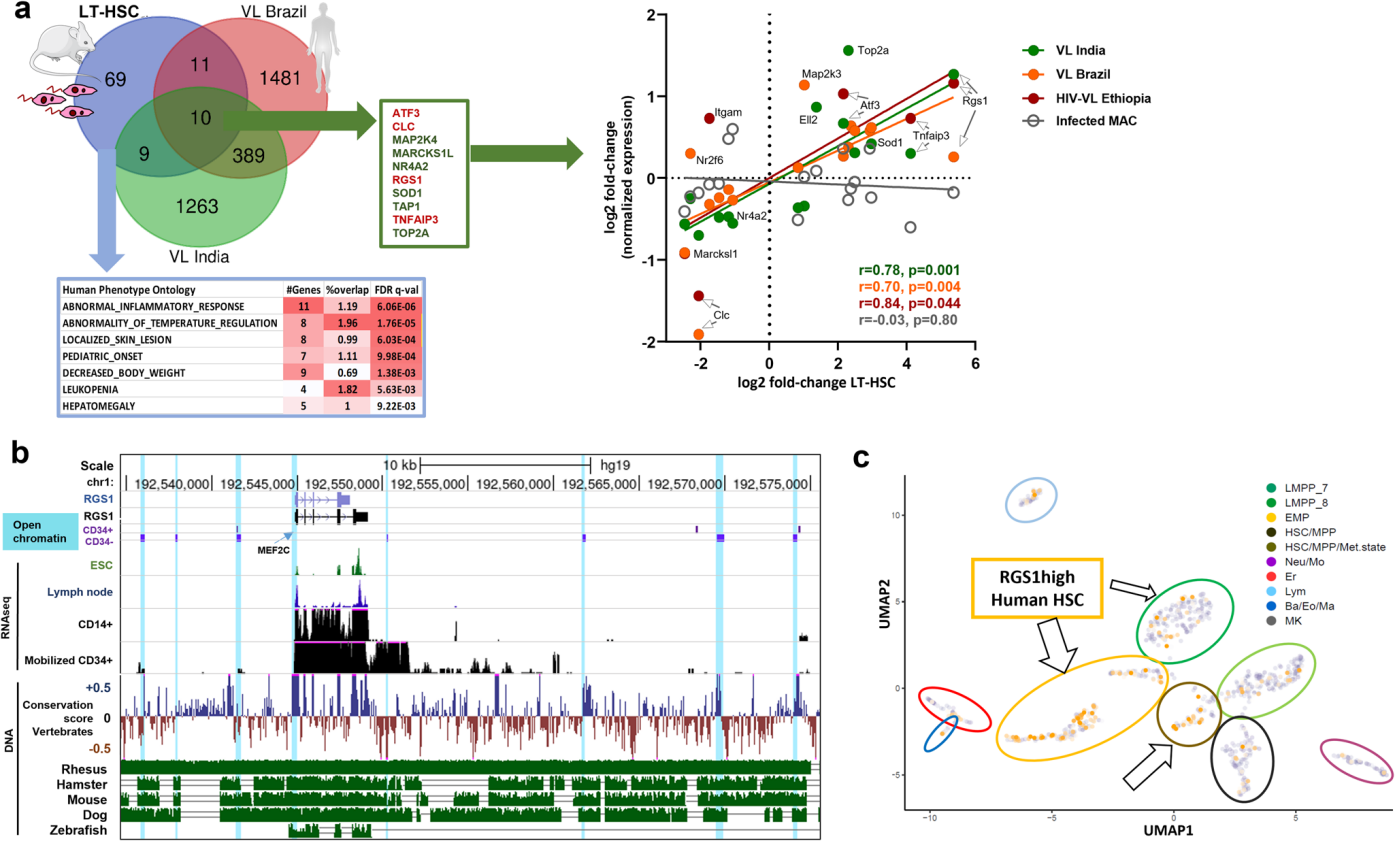
An evolutionary conserved StemLeish gene signature recapitulates cross-species epigenetic and transcriptional signatures of visceral leishmaniasis. **a** In vivo validation of StemLeish signature in three independent cohorts of human VL patients from Brazil, from India and HIV-VL co-infected patients from Ethiopia, all fold-changes of DEG from untreated patients with active disease *vs*. post-treatment blood samples. Venn diagram (upper left panel) shows overlap between murine and human signatures. Human Phenotype Ontology (MSigDb) enrichment analysis indicates the StemLeish signature phenocopies the complete clinical picture of human VL (lower left panel). Significant correlations (Spearman) between StemLeish and human VL DEG in all three cohorts (right panel). **b** Integrated epigenetic, transcriptomic and genomic analysis of the *Rgs1* locus, visualized in UCSC Gene browser. Open chromatin regions (DNase I Hypersensitive Sites) in purified CD34^+ ^HSC (indicated as CD34^+^) and HSC undergoing myeloid differentiation (indicated as CD34^−^) are indicated by light blue shades. Transcriptomic data (RNAseq) represent mapped reads present in purified CD14^+^ vs. purified mobilized CD34^+^ cells, with embryonic stem cells (ESC) and lymph node RNAseq data plotted for comparison. Genomic data show conservation score across 100 different vertebrate species (blue: conserved, brown: not conserved), with leishmaniasis and tuberculosis model species (rhesus macaques, mouse, hamster, dog, zebrafish) plotted individually (green panels below), as compared to the human reference genome. **c** Reanalysis of single-cell RNAseq data^50^ using UMAP (Unifold Mapping) revealed presence within purified human CD34^+^ cells of an *RGS1*^*high*^ phenotype in several progenitor subsets [Erythro-Myeloid Progenitors (EMP), Lymphoid/Myeloid Progenitors (LMPP7), HSC in a metabolically active state (HSC/Met. state)]. Arrows demonstrate clusters containing *RGS1*^*high*^ cells (orange vs. gray *RGS1*^*low*^ cells). Arrow sizes are proportional to the frequency of *RGS1*^*high*^ cells. Clipart used in diagram (**a**) was obtained from Servier Medical Art (https://smart.servier.com).

Following identification of the StemLeish gene signature, we next sought to investigate a possible presence of this signature in three independent cohorts of human visceral leishmaniasis (patients from Brazil^43^, from India^48^ and HIV co-infected VL patients from Ethiopia^49^) considering the fold-changes of DEG between untreated patients with active disease and post-treatment blood samples. The Venn diagram (Fig. 6a, upper left panel) illustrates the overlap between murine StemLeish and human VL signatures, with notable conservation of the *Rgs1*/*Tnfaip3*/*Atf3* signature. Detection of the StemLeish signature in blood of VL patients supports the notion that HSC migrate from the BM to the circulation during VL^47^. Human Phenotype Ontology (MSigDb) enrichment analysis reveals that the StemLeish signature phenocopies the complete clinical picture of human VL (Fig. 6a, lower left panel), with a significant cross-species correlation (Spearman) in the fold-changes of shared DEG (Fig. 6a, right panel), including in the cohort with HIV co-infection.

We also compiled existing epigenetic, transcriptional, and genomic data for the prominent StemLeish gene *Rgs1* (Fig. 6b), demonstrating similar epigenetic regulation of open chromatin (ATAC-Seq, DNAse I hypersensitive sites) in human undifferentiated CD34^+^ HSC vs. HSC induced towards myeloid differentiation. The open chromatin regions in differentiated HSC (indicated as CD34^−^) correspond to increased transcription (Fig. 6b) and are conserved in all currently used animal models of leishmaniasis (rhesus macaque, mouse, hamster, dog), but not in zebrafish. However, the enhancer region upstream of *Rgs1* (containing the MEF2C binding site) is highly conserved among all vertebrates. Similar correlations between epigenetic and transcriptomic profiles were obtained for the other prominent StemLeish genes (Supplementary Fig. 6). *Rgs1* transcription is generally very low in embryonic stem cells (Fig. 6b and Supplementary Fig. 5b) but reexamination of published single-cell RNAseq data^50^ from cord blood confirmed the presence of an *RGS1*^*high*^ phenotype in several human CD34^+^ HSC subsets. More specifically, *RGS1*^*high*^ cells were frequent among Erythro-Myeloid Precursors (EMP), Lymphoid/Myeloid Precursors (LMPP7), and HSC in a metabolically active state (Fig. 6c, HSC/Met. state).

## Discussion

Many infectious diseases are complicated by post-treatment clinical reactivation, and protective tissues or cellular niches have recently gained increasing awareness. A well-known example is the liver which can be colonized by dormant or hypnozoite stages of *P. vivax*. These stages are less susceptible for antimalarial therapies and can reactivate^17,51^. The adipose tissue has been described as a hiding place for *Trypanosoma* species, which may be less amenable for drug treatment due to a low tissue perfusion rate^16,52^. In the present study, the importance of the BM as a relapse-prone niche during VL was unveiled. For both the adipose tissue^53^ and the bone marrow^30^ low perfusion rates have been described. In addition, drug concentrations within an organ can diverge at a cellular level. Zhao et al.^54^ have shown that HSC can differently respond to chemotherapy based on their position in the BM and distance to the blood vessel. The possibility of the BM as sanctuary site was already indicated in 2014 by a rare clinical case of VL after allogeneic BM transplantation from an asymptomatic Portuguese donor^25^. Our data using 90 different antileishmanial compounds whereby various degrees of efficacy were achieved in the BM is considerable. Whereas several compounds prove to be highly effective in this niche, others were only poorly active. For instance, at effective doses for the liver, the 8-aminoquinoline analogs Tafenoquine and Sitamaquine cannot effectively penetrate the BM, which is in line with the rather poor/variable efficacy in Phase II clinical trials^55^.

Parasite replication at systemic sites such as the BM is characteristic for the progressive disease course of VL. Chronic *L. donovani* infection studies in mice revealed that emergency hematopoiesis is induced, a stress response that activates HSC and results in hematological alterations^19^. Besides *Leishmania*, the BM was identified as an antibiotic-protective niche for *M. tuberculosis* where it was shown that *Mtb* can infect CD271^+^ BM mesenchymal stem cells (MSC) in vitro and in vivo in the presence or absence of antibiotic pressure^56^. It was also demonstrated that even after prolonged treatment, *Mtb* survived in CD271^+^ MSCs^18^ as well as it replicates within HSCs^57^. Apart from MSCs and HSCs, the presence of *Mtb* in LT-HSC has been documented in both human and mouse latent TB infections^58,59^. Moreover, CD34^+^ LT-HSC were recently confirmed as the main in vivo reservoir of circulating mycobacteria in a large cohort of *M. tuberculosis* and *M. bovis* infected individuals^60^.

HSC are progenitor cells that continuously replenish all blood cell types and can be classified into two subsets according to their long-term or short-term self-renewal capacity. Long-term HSC (Lin^−^Sca1^+^cKit^+^CD48^−^CD150^+^) can regenerate over a longer period of time and will subsequently differentiate into short-term HSC (Lin^−^Sca1^+^cKit^+^CD48^−^CD150^−^) and multipotent progenitors (MPP2: Lin^−^Sca1^+^cKit^+^CD48^+^CD150^+^ and MPP3/4: Lin^−^Sca1^+^cKit^+^CD48^+^CD150^−^). MPP lack self-renewal capacity but can further differentiate in lineage-committed cells such as common lymphoid progenitors (CLP) or common myeloid progenitors (CMP)^61–64^. MSC (CD90.2^+^CD105^+^CD271^+^) are distinct from HSC as they shape the BM stroma by differentiating in adipocytes, osteoblasts, osteoclasts, fibroblasts, endothelial cells, and smooth muscle cells^65^. LT-HSC reside in the immune-privileged niche of the BM^27^ and have been characterized as relatively quiescent stem cells (in the G0 phase of cell cycle) with the capacity of self-renewal^31,66,67^. During chronic VL infection, most LT-HSC were found to have entered cell cycle (G0 to G1) correlating with functional exhaustion. More specifically, HSCs were skewed to differentiate into non-classical myeloid progenitors with a regulatory suppressor cell-like phenotype that is more permissive to parasite infection^21^. Modification of the host’s BM emergency response thus enables *Leishmania* to promote its own proliferation and allows continued infection. Lopes et al.^40^ recently described the importance of the BM stroma and showed that *L. infantum* is capable of infecting CD45^+^ BM cells and CD271^+^CD45^−^ MSC in vitro and in vivo. In a study by Pinto et al. that did not include a progenitor enrichment^19^, no infected LT-HSC were detected after 28 days of infection with Td-Tomato transgenic *L. donovani*. In contrast, an in vitro study by Carvalho-Gontijo et al.^68^ documented the presence of *L. infantum* in human CD34^+^ stem cells. According to Cotterell et al.^69^, CD34^+^ progenitor cells do not appear to be targets for *Leishmania* infection. An important difference between CD34^+^ progenitors and LT-HSC in mice, is that LT-HSC are CD34^−^, a phenotype already acknowledged for a few decades^19,70,71^. Despite the apparent controversy, our study further pinpointed the exact tropism of visceral *Leishmania* species in the various stem cell and progenitor subsets in vitro and in vivo, using specific enrichment steps. Parasite loads differed substantially in these cell types with single parasites in BM monocytes whereas macrophages and myeloblasts can harbor multiple^21^. In our study, mouse LT-HSC and human HSPC were found to accommodate excessive burdens of parasites. Our data also show that LT-HSC prominently harbor viable parasites after drug treatment in vitro and in vivo. It deserves emphasis that the detection of a fluorescent protein expressed by transgenic parasites revealed to be an unreliable indicator of viable parasite presence. The higher in vitro clearance from macrophages and the reduction of viable parasite to undetectable levels in Lin^+^ cells following in vivo treatment suggest that LT-HSC more than stromal macrophages play a prominent role in the process of treatment failure. This does not exclude the possible involvement of other sanctuary cells, such as the MPP2 cells that also retain viable parasites. Spatial information about host cell tropism and fate of released amastigotes and infected cells will be helpful to shed more light on the dynamic processes that occur just after treatment. It has already been described that some intrinsic properties of stem cells may provide opportunities for the pathogen to evade immune responses and drug action, e.g., by avoiding the induction of cytotoxic T cell responses and by enhanced drug efflux^31,40^. Although SSG efflux transport was more prominent in LT-HSC, cell-intrinsic differences of drug responsiveness are not limited to drug efflux and likely relate to the vast differences in intracellular amastigote burdens. Based on a large in vivo drug evaluation effort (Fig. 1d), drug discovery activities should preferably include this particular BM niche during early discovery phase. Drugs with favorable pharmacokinetic properties to target the BM would potentially be more effective in targeting the LT-HSC burdens to prevent persistence and post-treatment relapse.

Besides the implications for drug discovery, the fundamental biology of parasite interaction with these cells deserves further exploration. Transcriptomic profiling of infected LT-HSC and cross-species systems biology analysis of large publicly available datasets revealed a unique *Leishmania*-triggered gene signature in LT-HSC, StemLeish, containing several host genes not previously associated with either experimental or human leishmaniasis (*Rgs1*, *Twistnb*, *Ell2*, and *Vav1*). Although further mechanistic work will be necessary, our data mining approach shows that *Rgs1* and *Twistnb* are bona fide HSC genes and suggest they might function as antagonists of myeloid (neutrophil and macrophage) differentiation (Fig. 5e). During human VL infection, the signature is also apparent in the blood, which may be the result of HSC migration from the BM to the circulation^47^. The LT-HSC gene signature also phenocopies transcriptional blood profiles of human VL, including HIV-VL co-infection.

Another evolutionary conserved host response is the production of reactive oxygen and nitrogen species, a prominent and thoroughly regulated antileishmanial response of macrophages aimed at killing the parasite without damaging the host cell. These oxidative mechanisms are in part stimulated by phagocytosis and involve various signaling and effector molecules. NO is one of the major reactive species in macrophages produced by inducible nitric oxide synthase (iNOS, encoded by the *Nos2* gene)^72^ which mediates intracellular killing of *Leishmania*^73^. The effects of ROS are variable amongst *Leishmania* species: some are susceptible to their action (*L. donovani*^74^, *L. major*^75^), while others appear resistant (*L. guyanensis*^76^, *L. amazonensis*^77^). Interestingly, our experiments demonstrate substantially decreased levels of *Nos2* gene expression and of both NO and ROS in infected LT-HSC, creating a more hospitable environment for parasite survival and multiplication. Balanced ROS levels are known to be pivotal for naive LT-HSC to maintain stem cell function and hematopoietic homeostasis. Stem cells in the BM also reside in a relatively hypoxic environment where low ROS and NO levels sustain a quiescent state and support self-renewal capacity^78^. In the event of ROS induction, stem cell differentiation is triggered and can lead to HSC exhaustion^79^. Similarly, NO stimulation induces the expansion of HSCs and commitment to the myeloid progeny^80^.

In summary, our in vivo and ex vivo data demonstrate that LT-HSC in the BM represents an oxidative stress- and drug-resistant protective niche in VL. A unique LT-HSC StemLeish transcriptional profile was triggered by viscerotropic *Leishmania* infection, which recapitulated the in vivo blood profiles of human VL and includes potential therapeutic targets. Our results bring fundamental insights into host-pathogen interactions, but also highlight that drug discovery efforts will need to be tweaked to be effective against intracellular pathogens residing in this highly permissive host cell.

## Methods

### Ethical statement

The use of laboratory rodents was carried out in strict accordance with all mandatory guidelines (EU directives, including the Revised Directive, 2010/63/EU on the Protection of Animals used for Scientific Purposes that came into force on 01/01/2013, and the declaration of Helsinki in its latest version) and was approved by the Ethical Committee of the University of Antwerp, Belgium (UA-ECD 2019–04). Human bone marrow aspirate rest samples, obtained as a diagnostic sample without a written informed consent, were available for in vitro infection experiments following approval by the Committee of Medical Ethics UZA-UA (B3002021000027).

### *Leishmania* parasites

The *L. infantum* strain MHOM/FR/96/LEM3323, with an inherent Sb resistance, was obtained from an HIV-positive patient from the Languedoc area in Southern France and kindly provided by CNRL (Montpellier, France). The *L. donovani* strain MHOM/ET/67/L82 was isolated from an Ethiopian VL-patient. Both were modified to express bioluminescent (PpyRE9) and/or fluorescent (DsRed) reporter proteins (LEM3323 WT^*PpyRE9*^, LEM3323 WT^*PpyRE9/DsRed*^ and L82 WT^*PpyRE9/DsRed*^)^81^. Promastigotes were sub-cultured twice weekly at 25 °C in hemoflagellate-modified minimal essential medium (HOMEM, Gibco®), supplemented with 10 % inactivated fetal calf serum (iFCS), 200 mM L-glutamine, 16.5 mM NaHCO_3_, 40 mg/L adenine, 3 mg/L folic acid, 2 mg/L D-biotin, and 2.5 mg/L hemin. The number of passages was kept as low as possible to maintain parasite virulence.

### Laboratory animals

Female BALB/c mice (6–8 weeks old) and female golden hamsters (BW 90–97 g) were purchased from Janvier (Genest-Saint-Isle, France) and accommodated in individually ventilated cages in pathogen-free conditions. They were provided with food for laboratory rodents (Carfil, Arendonk, Belgium) and water *ad libitum*. Animals were subdivided into experimental groups based on simple randomization. Mice were kept in quarantine for at least 5 days before starting the experiment. Euthanasia was performed in CO_2_ chambers followed by cervical dislocation, and tissues were collected under aseptic conditions.

### Primary mouse cells

Mouse BM was collected from BALB/c mice using two distinct techniques, based on pilot studies comparing alternative methods in terms of yield and quality. For both techniques, mice were sacrificed, and hind legs aseptically removed. Isolated femurs and tibias were cleaned by removing soft tissue from the bone using 70% ethanol-soaked cloth and tweezers.

For the crushing technique, the protocol was adapted from Lo Celso and Scadden^82^. Briefly, bones were crushed with mortar and pestle in ammonium-chloride-potassium (ACK) buffer (0.15 M NH_4_Cl, 1.0 mM KHCO_3_, 0.1 mM Na_2_EDTA) for erythrocyte lysis. Single cell suspensions were obtained by filtering through MACS® SmartStrainers (100 μm, Miltenyi Biotec), centrifuged at 500 × *g* for 10 min (4 °C), and resuspended in phosphate-buffered saline (PBS) + 0.2% bovine serum albumin (BSA). For efficient depletion of mature lineage-positive hematopoietic cells and to specifically isolate the preferred lineage-negative cells (i.e., undifferentiated progenitor cells), the Direct Lineage Cell Depletion Kit (Miltenyi Biotec) was employed according to the manufacturer’s instructions. Following lineage depletion, cells were counted in PBS and resuspended in PBS + 0.2% BSA buffer to 2 × 10^7^ cells/mL. Cells were kept on ice during all procedures.

The centrifugation method was adjusted from the protocol described by Amend et al.^83^ and Dobson et al.^84^ and used for subsequent macrophage and dendritic cell differentiation. Briefly, a 0.5 mL microcentrifuge tube was perforated at the bottom with a 21 G needle and nested inside a 1.5 mL tube (both from Eppendorf). After collection of femurs and tibias, one proximal end (knee epiphysis) was cut-off and placed in the 0.5 mL tube. Nested tubes were centrifuged in a microcentrifuge at 10,000 × *g* for 15 sec. Both long bones became white and a large visual pellet was observed in the 1.5 mL tube. This pellet was then resuspended in ACK buffer for erythrocyte lysis.

To obtain BM-derived macrophages (BMDM), cells were centrifuged at 500 × *g* for 10 min at 4 °C, resuspended in Roswell Park Memorial Institute (RPMI) medium (Gibco®) and divided over Petri dishes (Starstedt) supplemented with BM medium [RPMI 1640 medium with 10% (v/v) iFCS, 1% non-essential amino acids (NEAA), 1% sodium pyruvate, 1% L-glutamine, 50 U/mL penicillin, 50 μg/mL streptomycin (all from Gibco®) and 15% L929 supernatant with M-CSF]. Following a 6-day incubation at 37 °C with 5% CO_2_, the macrophages were collected by replacing the BM medium with ice cold dissociation buffer [PBS with 1% 0.5 M ethylenediaminetetraacetic acid (EDTA) and 2% 1 M 4-(2-hydroxyethyl)-1-piperazine-ethanesulfonic acid (HEPES)]. After detachment, the macrophage cell suspension was centrifuged at 500 × *g* for 10 min and resuspended in RPMI medium. The number of macrophages was counted in PBS using a KOVA® counting chamber. Cells were seeded in a 96-well plate (3 × 10^4^ cells/well) or a 24-well plate (1 × 10^6^ cells/well) and incubated for 24 h at 37 °C with 5% CO_2_ to allow adherence of the BMDMs.

To differentiate BM cells into dendritic cells (BMDCs), cells were cultured in Petri dishes in DC medium [RPMI 1640 supplemented with 10% (v/v) iFCS, 2 mM Glutamax, 20 mM HEPES, 50 U/mL penicillin, 50 μg/mL streptomycin, 50 μM 2-mercaptoethanol (all from Gibco®) with 200 U/mL recombinant murine granulocyte-macrophage colony-stimulating factor (GM-CSF; Peprotech)], followed by a 9-day incubation at 37 °C with 5% CO_2_ during which the medium was refreshed twice. After 9 days of differentiation with GM-CSF, preheated DC medium was used to recover the semi-adherent BMDC fraction which is reported to contain most CD11c^+^ and MHC-II^hi^ DCs^85^. Following a 10 min centrifugation at 500 × *g*, cells were resuspended in DC medium and counted in PBS using a KOVA® counting chamber. Cells were then plated in a 24-well plate (1 × 10^6^ cells/well) and incubated at 37 °C with 5% CO_2_.

### Primary human BM cells

Human BM aspirate was obtained from the iliac crest using BD Vacutainer® Plastic K3EDTA Tubes, initially collected for diagnostics, and delivered as residual sample. The BM was subjected to erythrocyte lysis twice using ACK buffer. Single cell suspensions were obtained by filtering through MACS® SmartStrainers (100 μm, Miltenyi Biotec), centrifuged at 300 × *g* for 10 min (4 °C), and resuspended in PBS + 0.2% BSA. Cells were counted in PBS and diluted to a concentration of 2 × 10^7^ cells/mL for flow cytometric analysis. Cells were kept on ice during these procedures.

### In vitro and in vivo visceral *Leishmania* infections

Parasite density was assessed by counting parasites in PBS using a KOVA® counting chamber. For in vitro infections, cell monolayers were co-cultured with stationary-phase promastigotes of *L. infantum* or *L. donovani* at a multiplicity of infection (MOI) of 5 for a minimum of 24 h at 37 °C with 5% CO_2_. In some experiments, amastigotes were purified from 96 h infected macrophages and used to initiate infection at a MOI of 5. For in vivo infection, stationary-phase parasites were centrifuged for 10 min at 4000 × *g* (25 °C) and resuspended to 1 × 10^9^ parasites/mL in sterile RPMI medium. Mice were infected intravenously (i.v.) in the lateral tail vein with 1 × 10^8^ parasites in 100 µL of RPMI medium.

### In vivo bioluminescent imaging (BLI)

Animals were monitored using in vivo BLI at selected time points. Imaging was performed 3 min after intraperitoneal (i.p.) injection of 150 mg/kg D-Luciferin (Beetle Luciferin Potassium Salt, Promega) in the IVIS® Spectrum In Vivo Imaging System under 2.5% isoflurane inhalation anesthesia using 15 min exposure. Images were analyzed using LivingImage v4.3.1 software by drawing regions of interests (ROIs) around specific organs to quantify the luminescent signal as relative luminescence units (RLU).

### Paromomycin post-treatment relapse model

Paromomycin sulfate salt (PMM, Sigma-Aldrich) stock solution was dissolved in MilliQ® at a concentration of 70 mg/mL. Mice were infected i.v. with 1 × 10^8^ metacyclic promastigotes of LEM3323 WT^*PpyRE9*^. Starting from 3 days post-infection (dpi), mice were treated i.p. for five consecutive days with 350 mg/kg *s.i.d.* PMM.

### Compound evaluation in the Syrian golden hamster model

For all compounds historically evaluated and reported in this study (Supplementary Data 1), the following screening protocol was performed: hamsters were allocated to experimental units of 5 to 6 animals based on live body weight at the start of the experiment. *L. infantum* (MHOM/MA(BE)/67) amastigotes obtained from the spleens of heavily infected donor hamsters were purified using two centrifugation steps and diluted to prepare an infection inoculum containing 2 × 10^7^ amastigotes/100 μL PBS. The infection inoculum was administered intracardially (i.c.). Amastigote burdens in the different target organs (liver, spleen, BM) were determined 10 days after the last treatment (i.e., day 35 of the experiment). The organs of individual animals were weighed (except BM); impression smears were Giemsa-stained for microscopic evaluation of amastigote burden, expressed as LDU (= mean number of amastigotes/cell × organ weight in mg). A minimum of 500 nuclei was counted. Percentage reduction compared to the burdens in the vehicle-treated infected control animals (VIC) was used as a measure for drug activity.

### Promastigote back-transformation

BM cells were mechanically disrupted to release the intracellular amastigotes in HOMEM promastigote medium. At 25 °C, amastigotes readily transform back into proliferative promastigotes^34^.

### Intracellular NO and ROS staining

To detect NO and ROS, DAF-FM Diacetate (4-amino-5-methylamino-2′,7′-difluorofluorescein diacetate) and CM-H_2_DCFDA were used respectively (both from Thermo Fischer Scientific). Stock solutions were prepared at 5 mM in dimethyl sulfoxide (DMSO) stored at −20 °C, and diluted immediately prior to use. Cells were incubated in pre-warmed PBS containing either probe in a final working concentration of 5 µM. After 30 min at 37 °C with 5% CO_2_, the loading buffer was removed and cells were incubated for 15 minutes in dye-free medium at the same temperature to allow complete de-esterification of the intracellular diacetates. Cells were further processed for flow cytometry as described below.

### Flow cytometry

Cell suspensions (2 × 10^7^/mL concentration) were treated with FcɣR-blocking agent (anti-CD16/32, clone 2.4G2, BD Biosciences) for 15 min, followed by a washing step using 500 × *g* centrifugation and resuspension in PBS + 0.2% BSA buffer. Next, cells were incubated for 20 min at 4 °C with a mix of fluorescent conjugated anti-mouse antibodies (Supplementary Table 1) at optimized concentrations. DAPI Staining Solution (Miltenyi Biotec) was used to assess viability. Cells were measured by flow cytometry using MACSQuant® Analyzer 10 (Miltenyi Biotec) and analyses were performed using FlowLogic^TM^ Software (Miltenyi Biotec) following specific gating strategies (Supplementary Fig. 7 and Supplementary Table 3), confirmed with FMO controls.

### Fluorescence-activated cell sorting (FACS)

Cell suspensions were processed as above (flow cytometry) prior to cell sorting using a specific antibody mix (Supplementary Table 2). Cells were sorted using FACSMelody^TM^ (BD Bioscience) following specific gating strategies (Supplementary Fig. 7 and Supplementary Table 3), confirmed with FMO controls, and compensated using single stains. The quality of sorting was confirmed by analyzing post-sort samples.

### Epifluorescence and conventional microscopy

After cell sorting, LT-HSC were collected on slides by Cytospin^TM^ followed by Giemsa or fluorescence staining. For fluorescence, both LT-HSC and BM-derived macrophages were fixed using 2% paraformaldehyde for 15 min at ambient temperature, followed by two washes with PBS. Cell nuclei were stained with DAPI (4′, 6-diamidine-2′-phenylindole dihydrochloride) solution (Sigma-Aldrich) for 2 min at ambient temperature. Finally, a drop of DABCO (1,4-diazabicyclo[2.2.2]octane, mixture of 70% glycerol and PBS) was added, and analysis was performed using a fluorescence microscope (Zeiss Axio Observer Z1 epifluorescence microscope) with a ×63 oil objective lens and ZEN software 2.3 pro. For conventional microscopy, both LT-HSC and BM-derived macrophages were fixed using methanol and stained with Giemsa. To confirm intracellular amastigote multiplication and thus remove extracellular promastigotes as potentially confounding factor, media with horse serum (as opposed to bovine serum) was used according to^35^.

### RNA isolation

Total RNA was extracted from *L. infantum* infected mouse liver and spleen tissue using RNeasy Plus Mini kit, and BM using QIAamp® RNA Blood Mini kit (both from Qiagen), according to the manufacturer’s instruction. To exclude gDNA, an additional step using gDNA elimination columns (Monarch®) was performed. RNA samples were stored in aliquots at −80 °C until qPCR analysis.

### Real-time quantitative PCR

The primer sequences and RT-qPCR conditions for Spliced-Leader RNA (SL-RNA) were derived from^86^. The Step One Plus real-time PCR system (Applied Biosystems) was used for all real-time qPCR assays and melt curve analyses. RT-qPCR were performed in a 20 µL reaction mixture containing 10 µL of 2× SensiFAST SYBR Hi-ROX One-Step mix (Bioline), 1 µL of each primer (10 µM final concentration), 0.2 µL of reverse transcriptase, 0.4 µL of RNAse inhibitor, 4 µL of RNA template, and 3.4 µL of PCR water. Each assay was run in duplicate together with a blank control. Threshold cycles (Ct) were defined as the fractional cycle number at which the fluorescence passed the fixed threshold. Ct values were extracted by using the StepOne^TM^ software v2.3. The mRNA expression of each gene was calculated relative to the expression of the housekeeping gene (*Eef2* - *eukaryotic translation elongation factor 2)* (Supplementary Table 4). After normalization, the relative expression levels were analyzed by using GraphPad software 7.0.

### NanoString digital transcriptomics and bioinformatic analyses

Cell lysates (from approximately 10,000 LT-HSC or macrophages, triplicates for both infected and uninfected conditions) for nCounter (NanoString) analysis were prepared using RNeasy Lysis buffer (RLT buffer, Qiagen). Cell lysates were hybridized to unique capture/reporter pairs (50 bp each) targeting 784 transcripts (734 murine transcripts and 20 housekeeping genes present in the Nanostring Myeloid/Innate Immunity Panel, complemented with 20 murine and 10 *Leishmania spp*. transcripts from a customized panel) as well as 6 positive and 8 negative control probes (all from NanoString). 736 were above the detection threshold. Results were sequentially corrected for background (negative control probes), technical variation (positive control probes) and RNA content (housekeeping genes) using nSolver 4.0 (NanoString), followed by differential expression and pathway enrichment analysis (Rosalind (www.rosalind.bio), NetworkAnalyst 3.0^87^, MSigDb^88^), in conjunction with publicly available datasets (GEO (https://www.ncbi.nlm.nih.gov/geo/), ToppCell (toppcell.cchmc.org), ImmGen (www.immgen.org), ImmuCo^89^).

### Drug susceptibility determination

Used concentrations of reference drugs (PMM, Sb, MIL) were selected according to Maes et al.^36^. After 120 h of drug exposure at 37 °C and 5% CO_2_, both LT-HSC and BMDMs were fixed with methanol and stained with Giemsa to microscopically determine the number of intracellular amastigotes per nucleus, the percentage of infection, and the infection index. LT-HSC (3 × 10^4^ cells in 200 μL RPMI medium) were collected on slides by Cytospin^TM^ to enable processing for Giemsa staining. To confirm intracellular amastigote multiplication and thus remove extracellular promastigotes as potentially confounding factor, horse serum was used according to ref. ^35^.

### Efflux susceptibility assay

The efflux pump inhibitor verapamil (MDR and MRP inhibitor, Sigma-Aldrich) was formulated in 100% DMSO at 20 mM. The stock solution was further diluted in demineralized water and administered in a concentration of 8 µM based on^38^. In all assays, the final in-test concentration of DMSO did not exceed 1%. Dilutions of the reference drugs, PMM (120 µM), MIL (7.5 µM), and SSG (35 eq. Sb) were added either with or without verapamil. After 96 h of drug and inhibitor exposure at 37 °C and 5% CO_2_, both LT-HSC and BMDM were processed as described above, and reduction in amastigote burden in the treated cells was compared to that of the untreated control cells.

### Statistics and reproducibility

Statistical analyses were performed using GraphPad® Prism version 7.00 and version 9.0.1, ExcelSTAT and WEKA. Tests were considered statistically significant if *p* < 0.05. For the study of relapse using sub-curative paromomycin treatment, observations were reproducible in three independent experiments consisting of 3–6 BALB/c mice. BLI data were analyzed using a Mann–Whitney test (two-tailed). Organ burden reductions in golden Syrian hamsters treated with a broad set of antileishmanial test compounds (*n* = 90) were subjected to a Wilcoxon matched-pairs signed rank test (two-tailed). Parasite cellular tropism and posttreatment survival were determined by sorting various BM cell subsets combined with promastigote back-transformation. Statistical analysis was based on a combination of independent experiments (3 ≤ *n* ≤ 5) and 2-way ANOVA using titration endpoints with positive back-transformations as a measure of viable parasite presence. Susceptibility of LT-HSC in comparison with other cell subsets was quantified by flow cytometry at 3 time points (24, 48, and 72 hpi) in 2 ≤ *n* ≤ 5 individual infections for each cell type. Infection indices (DsRed MFI × % infection) were compared between LT-HSC and other BM cell subsets with 2-way ANOVA. Differences in amastigote multiplication inside LT-HSC and MPP*2* were determined at various time points, each using 3 ≤ *n* ≤ 5 individual infections, and applying a multiple *t* test (two-tailed). Infection parameters (MFI, % infection, and infection index) in LT-HSC and BM-derived dendritic cells and macrophages were compared using a Kruskal–Wallis test. Induction of ROS and NO were quantified in LT-HSC and BM-derived macrophages by flow cytometry at 24 and 48 hpi. The same observations were made in three independent experiments with *L. donovani* and *L. infantum*. MFI data from 155 ≤ *n* ≤ 16250 cellular events were subjected to multiple *t* tests (two-tailed) after outlier analysis (ROUT, *Q* = 1%). The impact of various reference drug treatments on *L. donovani* and *L. infantum* in LT-HSC and BM-derived macrophages were determined in two independent experiments by Giemsa staining. Statistical analysis was performed using a Mann–Whitney test on 45 ≤ *n* ≤ 250 cells for each condition. The effect of the efflux pump inhibitor verapamil on drug activity was similarly analyzed by Giemsa staining and a Mann–Whitney test, using data from three independent experiments and 60 ≤ *n* ≤ 100 cells for each condition.

The StemLeish gene signature (*Rgs1*/*Skil*/*Ell2*/*Cebpa*/*Twistnb*) was validated in large independent murine LT-HSC and macrophage datasets (ImmuCo; *n* = 645 and *n* = 785 mice respectively) and in three cohorts of human VL patients. Data expressed as RMA (Robust Microarray Average) or Log2-fold change were subjected to a Spearman’s correlation analysis to evaluate co-regulation in a quantitative manner across a large number of independent samples.

### Reporting summary

Further information on research design is available in the Nature Research Reporting Summary linked to this article.

## Supplementary information

Supplementary Information

Description of Additional Supplementary Files

Supplementary Data 1

Supplementary Data 2

Supplementary Data 3

Reporting Summary

## Data Availability

The authors declare that the data reported in this study are available within the paper and its supplementary information files. The source data of Figs. 1–6 and Supplementary Figs. 1–6 are provided in a separate data file (Supplementary Data 3). NCounter transcriptomics datasets have been at Gene Expression Omnibus (GEO accession GSE205452).

## Code availability

Transcript data were analyzed using nSolver 4.0 (NanoString), Rosalind (www.rosalind.bio), NetworkAnalyst 3.0^87^ and MSigDb^88^, in conjunction with publicly available datasets (GEO (https://www.ncbi.nlm.nih.gov/geo/), ToppCell (toppcell.cchmc.org), ImmGen (www.immgen.org) and ImmuCo^89^.

## References

[1] Burza S, Croft SL, Boelaert M (2018). Leishmaniasis. Lancet.

[2] Ready PD (2014). Epidemiology of visceral leishmaniasis. Clin. Epidemiol..

[3] Kamhawi S (2006). Phlebotomine sand flies and leishmania parasites: Friends or foes?. Trends Parasitol..

[4] Oliveira F, de Carvalho AM, de Oliveira CI (2013). Sand-fly saliva-leishmania-man: The trigger trio. Front. Immunol..

[5] Feijo D, Tiburcio R, Ampuero M, Brodskyn C, Tavares N (2016). Dendritic cells and leishmania infection: Adding layers of complexity to a complex disease. J. Immunol. Res..

[6] Martinez-Lopez M, Soto M, Iborra S, Sancho D (2018). Leishmania hijacks myeloid cells for immune escape. Front. Microbiol..

[7] Kedzierski, L. & Evans, K. J. Immune responses during cutaneous and visceral leishmaniasis. *Parasitology* **141**, 1–19 (2014). 10.1017/S003118201400095X 25075460

[8] Horrillo L (2019). Clinical aspects of visceral leishmaniasis caused by L. infantum in adults. Ten years of experience of the largest outbreak in Europe: What have we learned?. Parasit. Vectors.

[9] Rijal S (2013). Increasing failure of miltefosine in the treatment of Kala-azar in Nepal and the potential role of parasite drug resistance, reinfection, or noncompliance. Clin. Infect. Dis..

[10] Jha TK (1998). Randomised controlled trial of aminosidine (paromomycin) v sodium stibogluconate for treating visceral leishmaniasis in North Bihar, India. BMJ.

[11] Selvapandiyan A, Croft SL, Rijal S, Nakhasi HL, Ganguly NK (2019). Innovations for the elimination and control of visceral leishmaniasis. PLoS Negl. Trop. Dis..

[12] Alves, F. et al. Recent development of visceral leishmaniasis treatments: Successes, pitfalls, and perspectives. *Clin. Microbiol. Rev.*10.1128/CMR.00048-18 (2018). 10.1128/CMR.00048-18 PMC6148188 30158301

[13] Barrett MP, Kyle DE, Sibley LD, Radke JB, Tarleton RL (2019). Protozoan persister-like cells and drug treatment failure. Nat. Rev. Microbiol..

[14] Tanaka N, Ashour D, Dratz E, Halonen S (2016). Use of human induced pluripotent stem cell-derived neurons as a model for Cerebral Toxoplasmosis. Microbes Infect..

[15] Ferreira-da-Silva Mda F, Takacs AC, Barbosa HS, Gross U, Luder CG (2009). Primary skeletal muscle cells trigger spontaneous Toxoplasma gondii tachyzoite-to-bradyzoite conversion at higher rates than fibroblasts. Int. J. Med. Microbiol.

[16] Ferreira AV (2011). Evidence for Trypanosoma cruzi in adipose tissue in human chronic Chagas disease. Microbes Infect..

[17] Shanks GD, White NJ (2013). The activation of vivax malaria hypnozoites by infectious diseases. Lancet Infect. Dis..

[18] Beamer G, Major S, Das B, Campos-Neto A (2014). Bone marrow mesenchymal stem cells provide an antibiotic-protective niche for persistent viable Mycobacterium tuberculosis that survive antibiotic treatment. Am. J. Pathol..

[19] Pinto AI (2017). TNF signalling drives expansion of bone marrow CD4+ T cells responsible for HSC exhaustion in experimental visceral leishmaniasis. PLoS Pathog..

[20] Yarali N, Fisgin T, Duru F, Kara A (2002). Myelodysplastic features in visceral leishmaniasis. Am. J. Hematol..

[21] Abidin BM, Hammami A, Stager S, Heinonen KM (2017). Infection-adapted emergency hematopoiesis promotes visceral leishmaniasis. PLoS Pathog..

[22] Varma N, Naseem S (2010). Hematologic changes in visceral leishmaniasis/kala azar. Indian J. Hematol. Blood Transfus..

[23] Liu D, Uzonna JE (2012). The early interaction of Leishmania with macrophages and dendritic cells and its influence on the host immune response. Front. Cell Infect. Microbiol.

[24] Ali N, Hussain S (2014). Leishmania donovani bodies in bone marrow. Clin. Case Rep..

[25] Dantas Brito M (2014). Visceral leishmaniasis: a differential diagnosis to remember after bone marrow transplantation. Case Rep. Hematol..

[26] Gawade S, Nanaware M, Gokhale R, Adhav P (2012). Visceral leishmaniasis: A case report. Australas. Med. J..

[27] Ichiryu N, Fairchild PJ (2013). Immune privilege of stem cells. Methods Mol. Biol..

[28] Lima-Junior DS (2013). Inflammasome-derived IL-1beta production induces nitric oxide-mediated resistance to Leishmania. Nat. Med..

[29] Novais FO (2014). Human classical monocytes control the intracellular stage of Leishmania braziliensis by reactive oxygen species. J. Infect. Dis..

[30] Mu CF (2018). Targeted drug delivery for tumor therapy inside the bone marrow. Biomaterials.

[31] Ng AP, Alexander WS (2017). Haematopoietic stem cells: Past, present, and future. Cell Death Discov..

[32] Dittus C, Semmel D (2013). Leishmania amastigotes visualized on bone marrow aspirate in a leishmaniasis and HIV coinfected patient presenting with pancytopenia. Blood.

[33] Zhang G, Zhong J, Wang T, Zhong L (2020). A case of visceral leishmaniasis found by left oblique hernia: A case report. Exp. Ther. Med..

[34] Hendrickx S (2014). Experimental selection of paromomycin and miltefosine resistance in intracellular amastigotes of Leishmania donovani and L. infantum. Parasitol. Res..

[35] Tegazzini D (2016). A replicative in vitro assay for drug discovery against leishmania donovani. Antimicrob. Agents Chemother..

[36] Maes, L., Cos, P. & Croft, S. L. *Drug Resistance in Leishmania Parasites: Consequences, Molecular Mechanisms and Possible Treatments* (eds Ponte-Sucre, A., Diaz, E. & Padrón-Nieves, M.) 407–429 (Springer Vienna, 2013).

[37] Bunting KD (2002). ABC transporters as phenotypic markers and functional regulators of stem cells. Stem Cells.

[38] Van den Kerkhof, M. et al. Antileishmanial aminopyrazoles: Studies into mechanisms and stability of experimental drug resistance. *Antimicrob Agents Chemother*10.1128/AAC.00152-20 (2020). 10.1128/AAC.00152-20 PMC7449183 32601168

[39] Valiathan R, Dubey ML, Mahajan RC, Malla N (2006). Leishmania donovani: Effect of verapamil on in vitro susceptibility of promastigote and amastigote stages of Indian clinical isolates to sodium stibogluconate. Exp. Parasitol..

[40] Lopes CS, Daifalla N, Das B, Dias da Silva V, Campos-Neto A (2016). CD271+ mesenchymal stem cells as a possible infectious niche for leishmania infantum. PLoS One.

[41] Rodriguez NE, Chang HK, Wilson ME (2004). Novel program of macrophage gene expression induced by phagocytosis of Leishmania chagasi. Infect. Immun..

[42] Frade AF (2011). TGFB1 and IL8 gene polymorphisms and susceptibility to visceral leishmaniasis. Infect. Genet. Evol..

[43] Gardinassi LG, Garcia GR, Costa CH, Costa Silva V, de Miranda Santos IK (2016). Blood transcriptional profiling reveals immunological signatures of distinct states of infection of humans with leishmania infantum. PLoS Negl. Trop. Dis..

[44] Gatto M (2020). Transcriptional analysis of THP-1 cells infected with Leishmania infantum indicates no activation of the inflammasome platform. PLoS Negl. Trop. Dis..

[45] Khouri R (2009). IFN-beta impairs superoxide-dependent parasite killing in human macrophages: Evidence for a deleterious role of SOD1 in cutaneous leishmaniasis. J. Immunol..

[46] Khouri R (2010). DETC induces Leishmania parasite killing in human in vitro and murine in vivo models: A promising therapeutic alternative in Leishmaniasis. PLoS One.

[47] Cotterell SE, Engwerda CR, Kaye PM (2000). Enhanced hematopoietic activity accompanies parasite expansion in the spleen and bone marrow of mice infected with Leishmania donovani. Infect. Immun..

[48] Fakiola M (2019). Transcriptional blood signatures for active and amphotericin B treated visceral leishmaniasis in India. PLoS Negl. Trop. Dis..

[49] Adriaensen W (2020). Host transcriptomic signature as alternative test-of-cure in visceral leishmaniasis patients co-infected with HIV. EBioMedicine.

[50] Zheng S, Papalexi E, Butler A, Stephenson W, Satija R (2018). Molecular transitions in early progenitors during human cord blood hematopoiesis. Mol. Syst. Biol..

[51] Rishikesh K, Saravu K (2016). Primaquine treatment and relapse in Plasmodium vivax malaria. Pathog. Glob. Health.

[52] Trindade S (2016). Trypanosoma brucei parasites occupy and functionally adapt to the adipose tissue in mice. Cell Host Microbe.

[53] Frayn KN, Karpe F (2014). Regulation of human subcutaneous adipose tissue blood flow. Int. J. Obes..

[54] Zhao M (2019). N-Cadherin-expressing bone and marrow stromal progenitor cells maintain reserve hematopoietic stem cells. Cell Rep..

[55] Sundar S, Chakravarty J (2015). Investigational drugs for visceral leishmaniasis. Expert Opin. Investig. Drugs.

[56] Das B (2013). CD271(+) bone marrow mesenchymal stem cells may provide a niche for dormant Mycobacterium tuberculosis. Sci. Transl. Med..

[57] Berry MP (2010). An interferon-inducible neutrophil-driven blood transcriptional signature in human tuberculosis. Nature.

[58] Tornack J (2017). Human and mouse hematopoietic stem cells are a depot for dormant mycobacterium tuberculosis. PLoS One.

[59] Delgobo, M. et al. An evolutionary recent IFN/IL-6/CEBP axis is linked to monocyte expansion and tuberculosis severity in humans. *Elife*10.7554/eLife.47013 (2019). 10.7554/eLife.47013 PMC6819084 31637998

[60] Belay M (2021). Detection of Mycobacterium tuberculosis complex DNA in CD34-positive peripheral blood mononuclear cells of asymptomatic tuberculosis contacts: an observational study. Lancet Microbe.

[61] Grinenko T (2018). Hematopoietic stem cells can differentiate into restricted myeloid progenitors before cell division in mice. Nat. Commun..

[62] Ema H, Morita Y, Suda T (2014). Heterogeneity and hierarchy of hematopoietic stem cells. Exp. Hematol..

[63] Seita J, Weissman IL (2010). Hematopoietic stem cell: Self-renewal versus differentiation. Wiley Interdiscip. Rev. Syst. Biol. Med..

[64] Gomes, A. C., Saraiva, M. & Gomes, M. S. The bone marrow hematopoietic niche and its adaptation to infection. *Semin. Cell Dev. Biol*. 10.1016/j.semcdb.2020.05.014 (2020). 10.1016/j.semcdb.2020.05.014 32553581

[65] Pittenger MF (2019). Mesenchymal stem cell perspective: Cell biology to clinical progress. NPJ Regen. Med..

[66] Boulais PE, Frenette PS (2015). Making sense of hematopoietic stem cell niches. Blood.

[67] Morrison SJ, Scadden DT (2014). The bone marrow niche for haematopoietic stem cells. Nature.

[68] Carvalho-Gontijo R (2018). Infection of hematopoietic stem cells by Leishmania infantum increases erythropoiesis and alters the phenotypic and functional profiles of progeny. Cell Immunol..

[69] Cotterell SE, Engwerda CR, Kaye PM (2000). Leishmania donovani infection of bone marrow stromal macrophages selectively enhances myelopoiesis, by a mechanism involving GM-CSF and TNF-alpha. Blood.

[70] Challen GA, Boles N, Lin KK, Goodell MA (2009). Mouse hematopoietic stem cell identification and analysis. Cytom. A.

[71] Kiel MJ (2005). SLAM family receptors distinguish hematopoietic stem and progenitor cells and reveal endothelial niches for stem cells. Cell.

[72] Rossi M, Fasel N (2018). How to master the host immune system? Leishmania parasites have the solutions!. Int. Immunol..

[73] Sarkar A (2011). Monitoring of intracellular nitric oxide in leishmaniasis: its applicability in patients with visceral leishmaniasis. Cytom. A.

[74] Murray HW, Nathan CF (1999). Macrophage microbicidal mechanisms in vivo: reactive nitrogen versus oxygen intermediates in the killing of intracellular visceral Leishmania donovani. J. Exp. Med..

[75] Blos M (2003). Organ-specific and stage-dependent control of Leishmania major infection by inducible nitric oxide synthase and phagocyte NADPH oxidase. Eur. J. Immunol..

[76] Sousa-Franco J (2006). Infection-induced respiratory burst in BALB/c macrophages kills Leishmania guyanensis amastigotes through apoptosis: Possible involvement in resistance to cutaneous leishmaniasis. Microbes Infect..

[77] Roma EH (2016). Impact of reactive oxygen species (ROS) on the control of parasite loads and inflammation in Leishmania amazonensis infection. Parasit. Vectors.

[78] Beltran-Povea A (2015). Role of nitric oxide in the maintenance of pluripotency and regulation of the hypoxia response in stem cells. World J. Stem Cells.

[79] Ludin A (2014). Reactive oxygen species regulate hematopoietic stem cell self-renewal, migration, and development, as well as their bone marrow microenvironment. Antioxid. Redox Signal.

[80] Nogueira-Pedro A (2014). Nitric oxide-induced murine hematopoietic stem cell fate involves multiple signaling proteins, gene expression, and redox modulation. Stem Cells.

[81] Bulte D (2021). Miltefosine enhances infectivity of a miltefosine-resistant Leishmania infantum strain by attenuating its innate immune recognition. PLoS Negl. Trop. Dis..

[82] Lo Celso, C. & Scadden, D. Isolation and transplantation of hematopoietic stem cells (HSCs). *J. Vis. Exp*. 10.3791/157 (2007). 10.3791/157 PMC2532946 18830434

[83] Amend, S. R., Valkenburg, K. C. & Pienta, K. J. Murine hind limb long bone dissection and bone marrow isolation. *J. Vis. Exp*. 10.3791/53936 (2016). 10.3791/53936 PMC4941920 27168390

[84] Dobson KR, Reading L, Haberey M, Marine X, Scutt A (1999). Centrifugal isolation of bone marrow from bone: An improved method for the recovery and quantitation of bone marrow osteoprogenitor cells from rat tibiae and femurae. Calcif. Tissue Int..

[85] Inaba K (1992). Generation of large numbers of dendritic cells from mouse bone marrow cultures supplemented with granulocyte/macrophage colony-stimulating factor. J. Exp. Med..

[86] Eberhardt E (2018). Evaluation of a pan-leishmania spliced-leader RNA detection method in human blood and experimentally infected Syrian golden hamsters. J. Mol. Diagn..

[87] Zhou G (2019). NetworkAnalyst 3.0: A visual analytics platform for comprehensive gene expression profiling and meta-analysis. Nucleic Acids Res..

[88] Subramanian A (2005). Gene set enrichment analysis: A knowledge-based approach for interpreting genome-wide expression profiles. Proc. Natl Acad. Sci. USA.

[89] Wang P (2015). ImmuCo: A database of gene co-expression in immune cells. Nucleic Acids Res..

