# Supplementary material for: Long-term hematopoietic stem cells as a parasite niche during treatment failure in visceral leishmaniasis
Source: Commun Biol. 2022 Jun 25;5:626. doi: 10.1038/s42003-022-03591-7 (PMC9233693; doi:10.1038/s42003-022-03591-7)
Supplement: Supplementary file 1 — Supplementary Information [file 42003_2022_3591_MOESM1_ESM.pdf]

Long-term hematopoietic stem cells as a parasite niche during treatment failure in visceral leishmaniasis

Laura Dirkx[1], Sarah Hendrickx[1], Margot Merlot[1], Dimitri Bulté[1], Marick Starick[2,3], Jessy Elst[4], André Bafica[3], Didier G. Ebo[4], Louis Maes[1], Johan Van Weyenbergh[2], Guy Caljon[1]*

[1]Laboratory of Microbiology, Parasitology and Hygiene (LMPH), Infla-Med Centre of Excellence, University of Antwerp, Antwerp, Belgium.

[2]Clinical and Epidemiological Virology, Department of Microbiology, Immunology and Transplantation, Rega Institute of Medical Research, KU Leuven, Leuven, Belgium.

[3]Laboratory of Immunobiology, Department of Microbiology, Immunology and Parasitology Federal University of Santa Catarina, Florianopolis, Brazil.

[4]Department of Immunology – Allergology – Rheumatology, Faculty of Medicine and Health Science and the Infla-Med Centre of Excellence, University of Antwerp, Antwerp University Hospital, Antwerp, Belgium.

Supplementary information

Supplementary Data 1: List of compounds and their dose tested in Syrian golden hamsters. Percentage of recovery (%R), single injection dose (SID), twice daily injection dose (BID).

Supplementary Data 2: Differential LT-HSC genes and systems biology analyses to evaluate enrichment of hallmark gene sets, pathways, transcription factor motifs, cell type enrichment and cross-pathology overlap.

Supplementary Data 3: The source data of Figures 1-6 and Supplementary Figures 1-6.

Supplementary figures

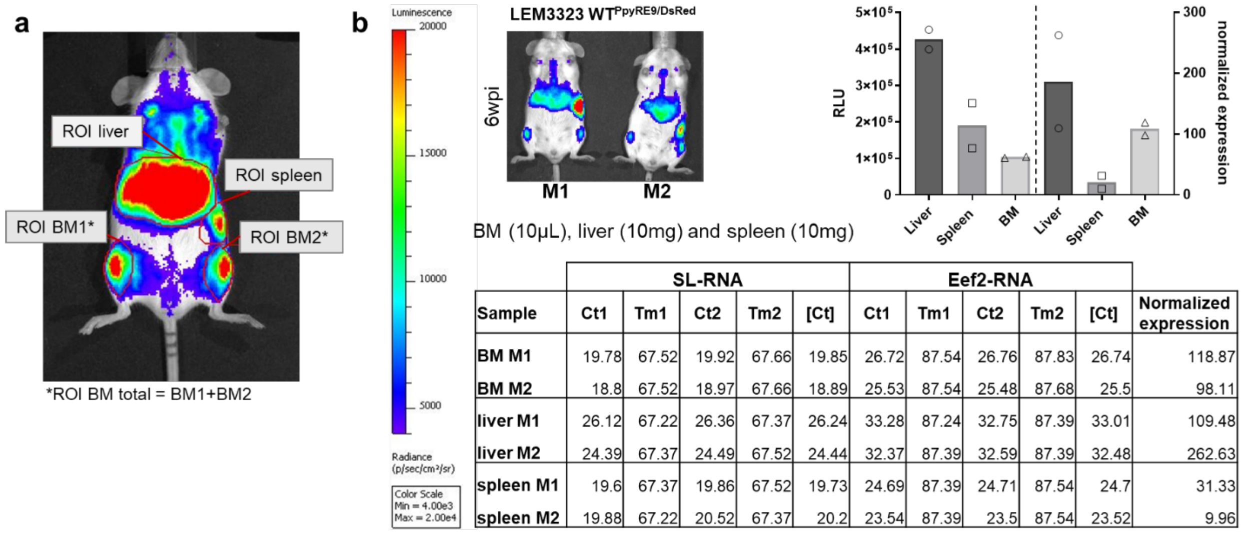

	SL-RNA					Eef2-RNA					
Sample	Ct1	Tm1	Ct2	Tm2	[Ct]	Ct1	Tm1	Ct2	Tm2	[Ct]	Normalized expression
BM M1	19.78	67.52	19.92	67.66	19.85	26.72	87.54	26.76	87.83	26.74	118.87
BM M2	18.8	67.52	18.97	67.66	18.89	25.53	87.54	25.48	87.68	25.5	98.11
liver M1	26.12	67.22	26.36	67.37	26.24	33.28	87.24	32.75	87.39	33.01	109.48
liver M2	24.39	67.37	24.49	67.52	24.44	32.37	87.39	32.59	87.39	32.48	262.63
spleen M1	19.6	67.37	19.86	67.52	19.73	24.69	87.39	24.71	87.54	24.7	31.33
spleen M2	19.88	67.22	20.52	67.37	20.2	23.54	87.39	23.5	87.54	23.52	9.96

Supplementary Figure 1. (a) regions of interest (ROI) to calculate relative luminescence units. **(b)** Quantitative analysis of BLI imaging using RT-qPCR shows an underestimation of BM infection. Representative example of mice infected for 6 weeks (6wpi) with LEM3323 WT$^{PpyRE9/DsRed}$ imaged with BLI and subsequently sacrificed to perform RT-qPCR on the liver, spleen and BM. Normalized expression based on SL-RNA, which represents the parasite RNA, and Eef2 as the control for mouse RNA. Results are based on two independent repeats, 2 BALB/c mice per group.

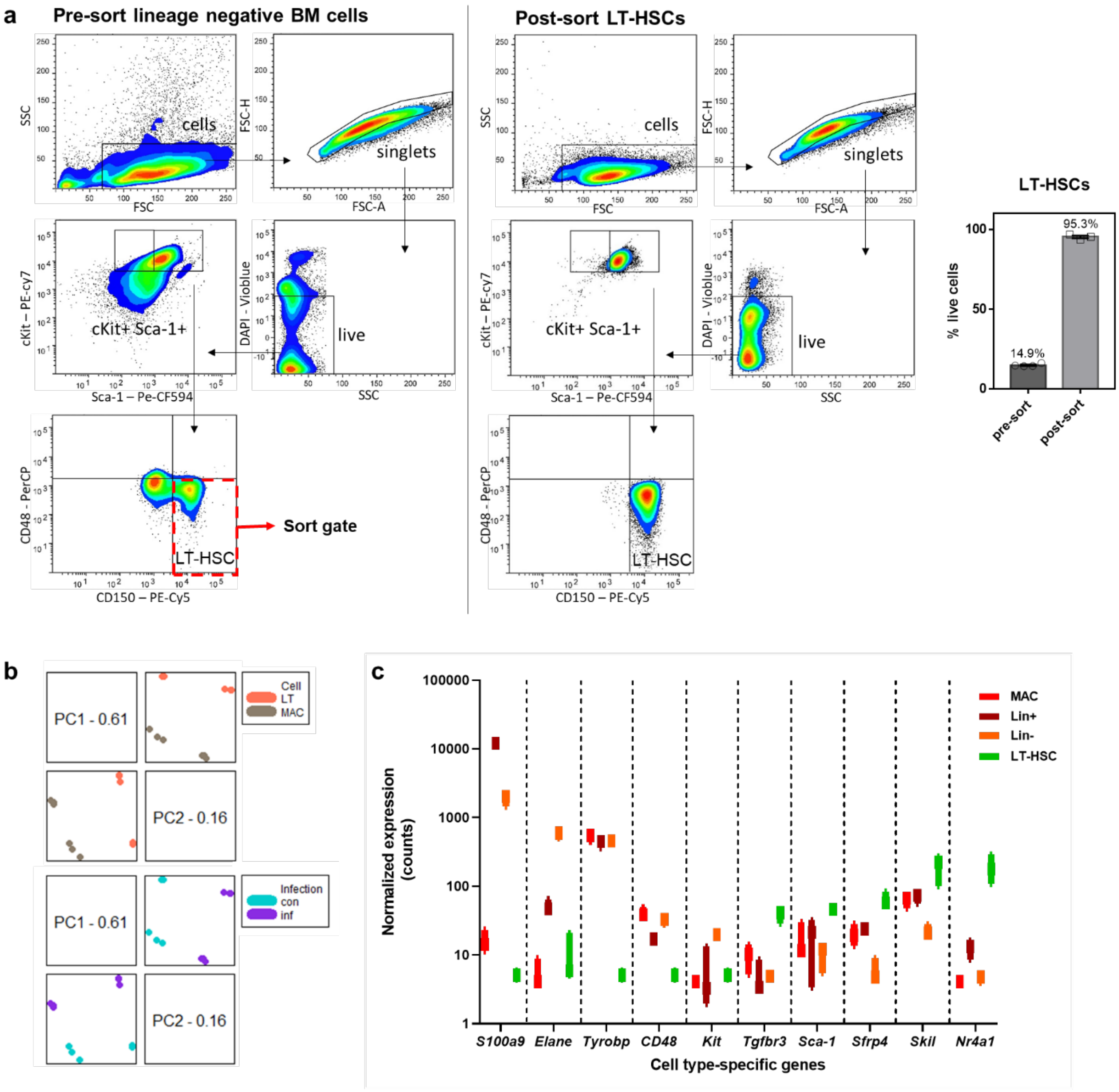

Supplementary Figure 2. Purity assessment of post-sort LT-HSC. (a) Lineage depleted BM of naive BALB/c mice was sorted for LT-HSC and re-measured to assess the purity of sorting with FACSMelody. **(b)** PCA analysis of the nCounter digital transcriptomics data revealing distant clustering of the LT-HSC and macrophage samples, confirming purity of the sorted samples. **(c)** Quantification of post-sorting purity using cell type-specific transcripts for Lin-, MAC, Lin+ and LT-HSC, including established (*S100a9*, *Elane*, *CD48*, *Kit*) and markers identified by nCounter analysis (*Tyrobp*, *Tgfbr3*, *Sfrp4*, *Skil*, *Nr4a1*). Based on the 7 most significant transcripts, the maximum contamination of purified LT-HSC with MAC, Lin+ or Lin- cells was 0.0009%, 0.001% and 0.8%, respectively.

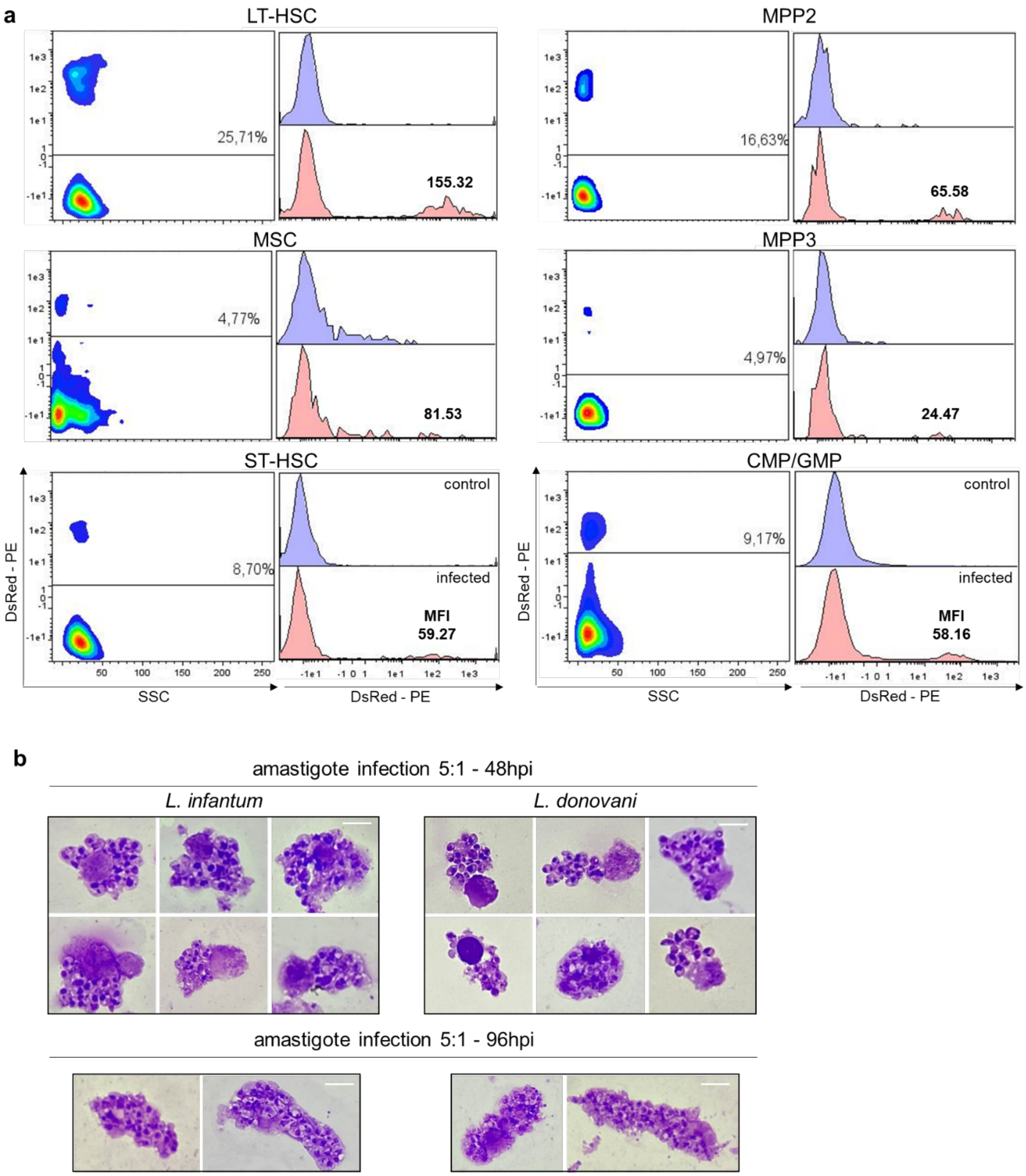

Supplementary Figure 3. (a) DsRed plots of all lineage negative BM subsets and histogram of representative result for Figure 3. Percentage of infection and MFI is provided on plots. Median fluorescence intensity (MFI). **(b)** Confirmation of amastigote infection of LT-HSC, both 48 and 96 hpi, using both *L. infantum* (LEM3323 WT$^{PpyRE9/DsRed}$) and *L. donovani* (Ldl82 WT$^{PpyRE9/DsRed}$) amastigotes purified from peritoneal macrophages 96 hpi. Scale bar = 10 µm.

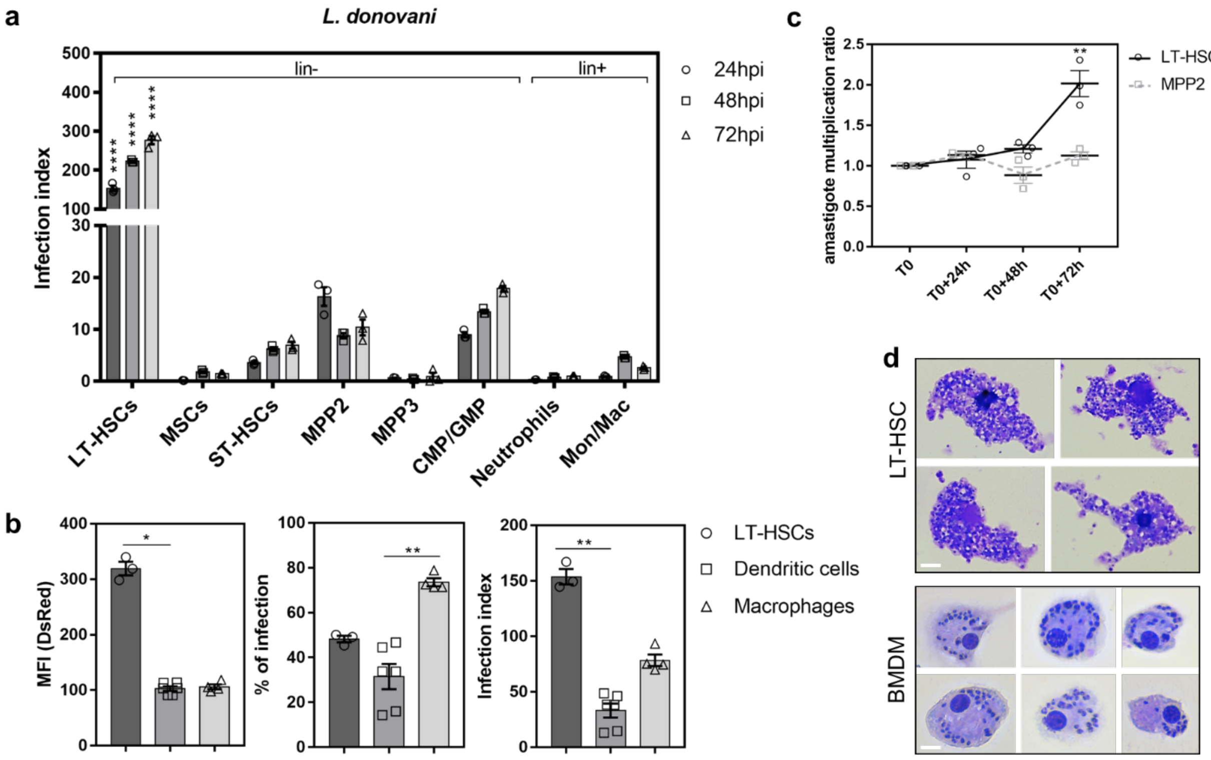

Supplementary Figure 4. LT-HSC are most susceptible to VL infection *ex vivo*. (a) *Ex vivo* Ldl82 $WT^{PpyRE9/DsRed}$ infection of lineage depleted BM collected from BALB/c mice: infection index representing DsRed MFI × % of infection. Statistical comparisons were made between LT-HSC and all other groups, for each time point. **(b)** Evolution over time of the intracellular parasite burden (amastigote multiplication ratio) in two BM cell subsets: LT-HSC and MPP2s. **(c)** Comparison of MFI, % infection and infection index between BM-derived dendritic cells and macrophages and LT-HSC, **(d)** Giemsa staining of Lld82 $WT^{PpyRE9/DsRed}$ infected BM-derived macrophages and sorted LT-HSC 120 hpi. Scale bar = 10 µm. **(a-d)** Days post-infection (dpi), median fluorescence intensity (MFI), mesenchymal stem cell (MSC), long-term hematopoietic stem cell (LT-HSC), short-term hematopoietic stem cell (ST-HSC), multipotent progenitor (MPP), common myeloid progenitor (CMP), granulocyte-monocyte progenitor (GMP), monocyte/macrophage (Mon/Mac). Results are shown as mean ± SEM and are based on at least three independent repeats ($3 \leq n \leq 6$). Statistical significance was found with two-tailed tests, *i.e.* 2-way ANOVA in (**a**), Kruskal-Wallis in (**b**), and multiple t test in (**d**). *p <0.05, **p <0.01, ****p <0.0001.

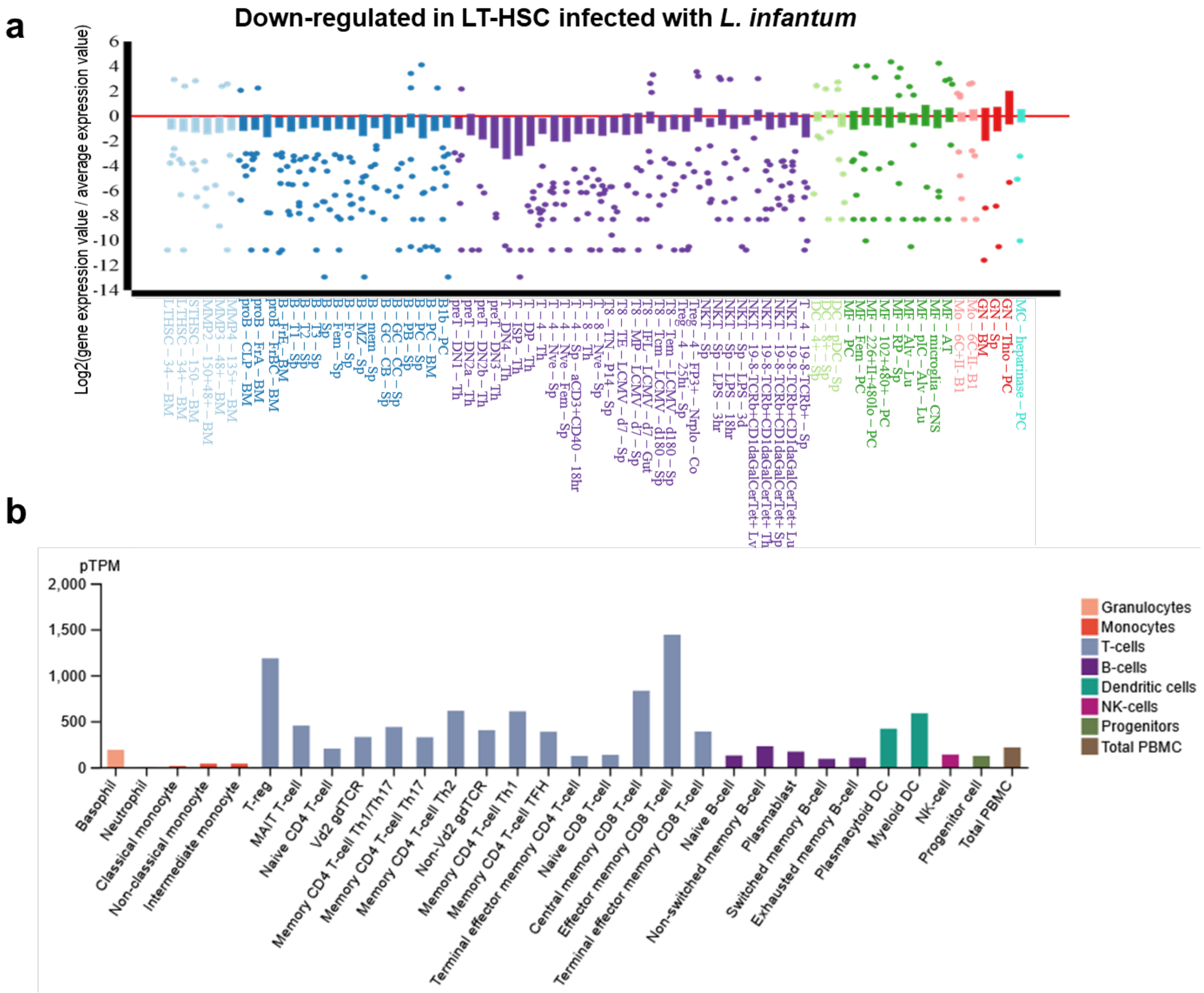

Supplementary Figure 5. Systems biology analysis of DEG in LT-HSC. (a) Genes down-regulated in LT-HSC upon infection with *L. infantum* are not significantly over- or under-expressed in any current known murine leukocyte subset in the exhaustive ImmGen database. **(b)** *Rgs1* mRNA is detectable in purified human progenitor (CD34+) and total PBMCS, and highly expressed in specific mature leukocyte subsets (Tregs, several CD4 memory subsets, CD8 central/effector memory cells, plasmacytoid and myeloid DCs).

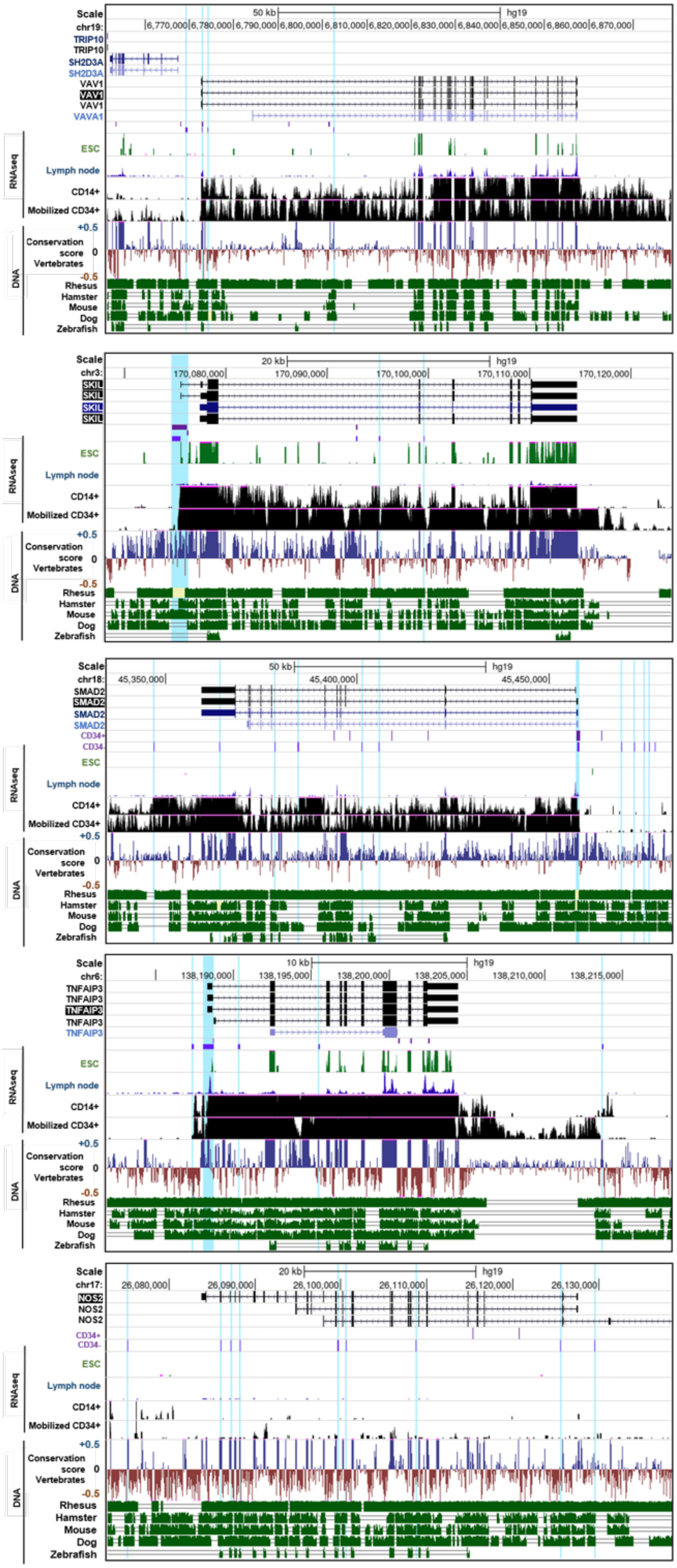
Scale
chr19:
50 kb
hg19
6,770,000
6,780,000
6,790,000
6,800,000
6,810,000
6,820,000
6,830,000
6,840,000
6,850,000
6,860,000
6,870,000
TRIP10
TRIP10
SH2D3A
SH2D3A
VAV1
VAV1
VAV1
VAVA1
RNAseq
ESC
Lymph node
CD14+
Mobilized CD34+
DNA
+0.5
Conservation score Vertebrates
0
-0.5
Rhesus
Hamster
Mouse
Dog
Zebrafish
Scale
chr3:
20 kb
hg19
170,080,000
170,090,000
170,100,000
170,110,000
170,120,000
SKIL
SKIL
SKIL
SKIL
RNAseq
ESC
Lymph node
CD14+
Mobilized CD34+
DNA
+0.5
Conservation score Vertebrates
0
-0.5
Rhesus
Hamster
Mouse
Dog
Zebrafish
Scale
chr18:
50 kb
hg19
45,350,000
45,400,000
45,450,000
SMAD2
SMAD2
SMAD2
SMAD2
CD34+
CD34-
RNAseq
ESC
Lymph node
CD14+
Mobilized CD34+
DNA
+0.5
Conservation score Vertebrates
0
-0.5
Rhesus
Hamster
Mouse
Dog
Zebrafish
Scale
chr6:
10 kb
hg19
138,190,000
138,195,000
138,200,000
138,205,000
138,210,000
138,215,000
TNFAIP3
TNFAIP3
TNFAIP3
TNFAIP3
TNFAIP3
RNAseq
ESC
Lymph node
CD14+
Mobilized CD34+
DNA
+0.5
Conservation score Vertebrates
0
-0.5
Rhesus
Hamster
Mouse
Dog
Zebrafish
Scale
chr17:
20 kb
hg19
26,080,000
26,090,000
26,100,000
26,110,000
26,120,000
26,130,000
NOS2
NOS2
NOS2
CD34+
CD34-
RNAseq
ESC
Lymph node
CD14+
Mobilized CD34+
DNA
+0.5
Conservation score Vertebrates
0
-0.5
Rhesus
Hamster
Mouse
Dog
Zebrafish

Supplementary Figure 6. StemLeish gene epigenomic/transcriptional regulation and genomic conservation. Integrated epigenetic, transcriptomic and genomic analysis of the *Vav1*, *Skil*, *Smad2*, *Tnfaip3* and *Nos2* loci, visualized in UCSC Gene browser. Open chromatin regions (DNase I Hypersensitive Sites) in purified CD34+ HSC (indicated as CD34+) and HSC undergoing myeloid differentiation (indicated as CD34-) are indicated by light blue shades. Transcriptomic data (RNAseq) represent mapped reads present in purified CD14+ *vs*. purified mobilized CD34+ cells, with embryonic stem cells (ESC) and lymph node RNAseq data plotted for comparison. Genomic conservation score across 100 different vertebrate species (blue: conserved, brown: not conserved), with leishmaniasis model species (rhesus macaques, mouse, hamster, dog) plotted individually (green panels below), as compared to the human reference genome.

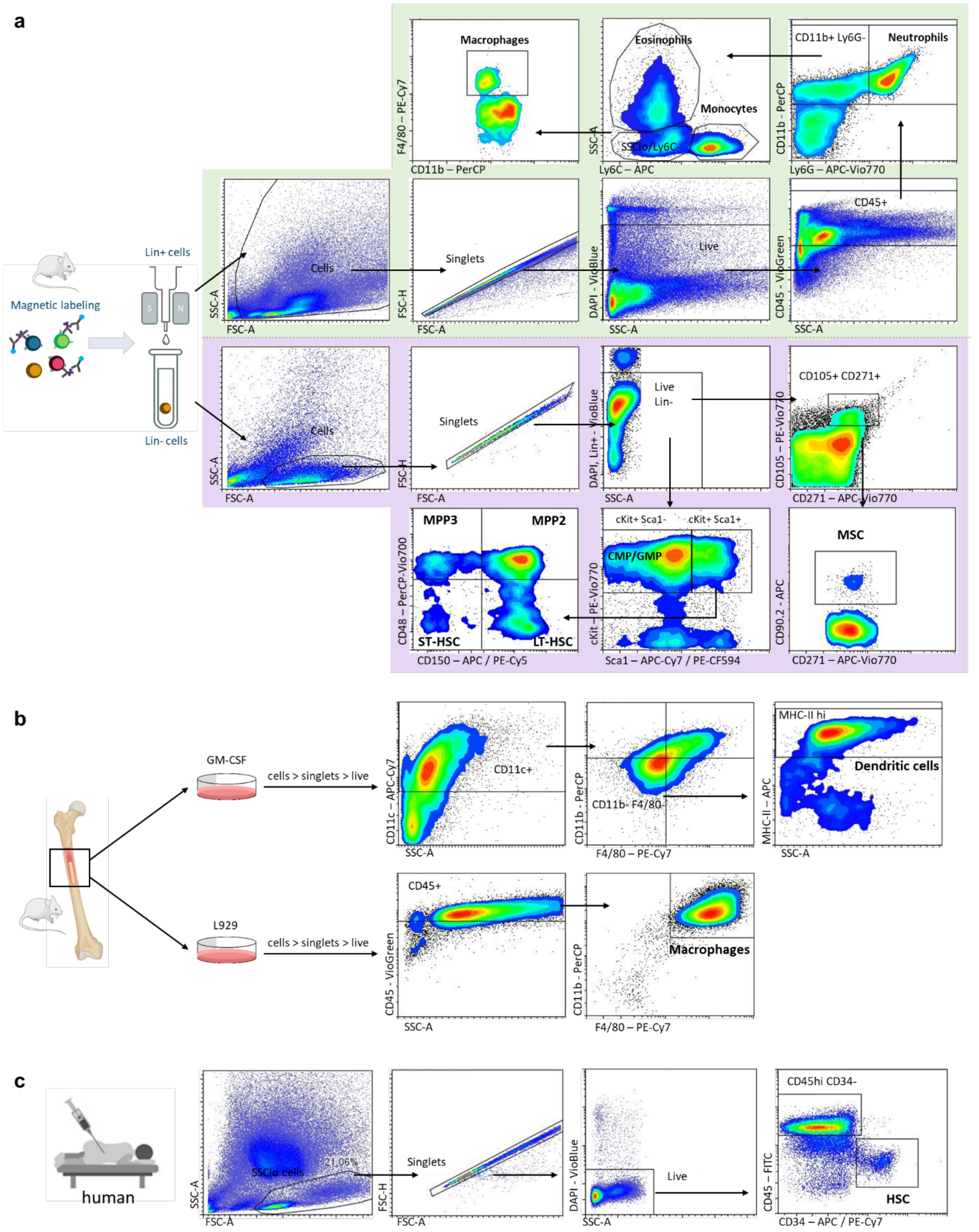

Supplementary Figure 7. Flow cytometry gating strategy. (a) BM cells were separated into lineage positive (Lin+) and negative (Lin-) cells using negative magnetic sorting. For both fractions, a cell gate was selected in a SSC-A versus FSC-A plot, followed by a gating on singlets in a FSC-H versus FSC-A plot. Lin+ live (DAPI-) CD45^{+} cells were further

characterized as neutrophils (CD11b+ Ly-6G+), monocytes (Ly-6Chi) and macrophages (SSC^{lo}, Ly-6C-, F4/80+). Lin- live (DAPI-) cells were either plotted as cKit versus Sca1 or as CD105 versus CD271 to allow identification of hematopoietic stem cells or mesenchymal stem cells, respectively. cKit+Sca1+ cells were further characterized as LT-HSC (CD48- CD150+), ST-HSCs (CD48- CD150-), MPP2 (CD48+ CD150+), and MPP3 (CD48+ CD150-). cKit+Sca1- cells were identified as CMP/GMP. CD105+CD271+ cells were further characterized as MSCs (CD90.2+). **(b)** BM cells were differentiated into dendritic cells or macrophages using GM-CSF or L929, respectively. For both subsets, a cell gate was selected in a SSC-A versus FSC-A plot, followed by gating on singlets in a FSC-H versus FSC-A plot and a live (DAPI-) selection as in (a). $CD45^{+}$ cells were further characterized as macrophages (CD11b+ F4/80+), and CD11c+ cells as dendritic cells (CD11b- F4/80- $MHC\text{-}II^{hi}$). **(c)** Human BM aspirate was treated twice with erythrocyte lysis buffer, a SSC low cell gate was selected in a SSC-A versus FSC-A plot, followed by gating on singlets in a FSC-H versus FSC-A plot. Live (DAPI-) cells were further characterized as HSCs ($CD45^{lo}$ CD34+) and $CD45^{hi}$ cells ($CD45^{hi}$ CD34-). Clipart was obtained from Servier Medical Art (https://smart.servier.com) and Biorender.com.

Supplementary Table 1. Cell markers to differentiate mouse BM cells by flow cytometry using MACSQuant® Analyzer 10. Abbreviations: long-term (LT) and short-term (ST) hematopoietic stem cell (HSC), multipotent progenitor (MPP), common myeloid progenitor (CMP), granulocyte monocyte progenitor (GMP).

Cell surface marker	Fluorophore	Clone N°	Manufacturer	Dilution
LT-HSC, ST-HSC, MPP2, MPP3, CMP, GMP				
cKit	PE-Vio770	REA791	Miltenyi Biotec	1:50
CD150	APC	REA299	Miltenyi Biotec	1:11
CD48	PerCP-Vio700	HM48-1	Miltenyi Biotec	1:100
CD34	FITC	REA383	Miltenyi Biotec	1:50
Ter119	VioBlue	Ter-119	Miltenyi Biotec	1:11
Gr-1	VioBlue	REA810	Miltenyi Biotec	1:500
CD11b	VioBlue	REA592	Miltenyi Biotec	1:500
TCR-β	VioBlue	REA318	Miltenyi Biotec	1:100
B220	VioBlue	REA755	Miltenyi Biotec	1:500
CD335	VioBlue	REA815	Miltenyi Biotec	1:50
Sca-1	APC-Cy7	D7	BD Biosciences	1:40
Monocytes, macrophages, neutrophils, eosinophils				
Ly6C	APC	AL-21	BD Biosciences	1:200
Ly6G	APC-Vio770	REA526	Miltenyi Biotec	1:100
CD11b	PerCP	M1/70	BioLegend®	1:400
F4/80	PE-Cy7	BM8	BioLegend®	1:200
CD45	VioGreen	REA737	Miltenyi Biotec	1:200
Mesenchymal stem cells				
CD105	PE-Vio770	REA1058	Miltenyi Biotec	1:50
CD271	APC-Vio770	REA648	Miltenyi Biotec	1:11
CD34	FITC	REA383	Miltenyi Biotec	1:50
Ter119	VioBlue	Ter-119	Miltenyi Biotec	1:11
Gr-1	VioBlue	REA810	Miltenyi Biotec	1:500
CD11b	VioBlue	REA592	Miltenyi Biotec	1:500
TCR-β	VioBlue	REA318	Miltenyi Biotec	1:100
B220	VioBlue	REA755	Miltenyi Biotec	1:500
CD335	VioBlue	REA815	Miltenyi Biotec	1:50
Dendritic cells				
CD11b	PerCP	M1/70	BioLegend®	1:400
F4/80	PE-Cy7	BM8	BioLegend®	1:200
CD11c	APC-Cy7	REA791	Miltenyi Biotec	1:100
MHC-II	APC	M5/114.15.2	Miltenyi Biotec	1:100
Human HSC and CD45^{hi} cells				
CD45	FITC	REA747	Miltenyi Biotec	1:140
CD34	APC	581	BioLegend®	1:20

Supplementary Table 2. Cell markers to differentiate BM cells by flow cytometry using FACSMelody™. Abbreviations: long-term (LT) and short-term (ST) hematopoietic stem cell (HSC), multipotent progenitor (MPP), common myeloid progenitor (CMP), granulocyte monocyte progenitor (GMP).

Cell surface marker	Fluorophore	Clone N°	Manufacturer	Dilution
Mouse LT-HSC, ST-HSC, MPP2, MPP3, CMP, GMP				
cKit	PE-Vio770	REA791	Miltenyi Biotec	1:50
CD150	PE-Cy5	TC15-12F12.2	BioLegend®	1:80
CD48	PerCP-Vio700	HM48-1	Miltenyi Biotec	1:100
CD34	FITC	REA383	Miltenyi Biotec	1:50
Ter119	VioBlue	Ter-119	Miltenyi Biotec	1:11
Gr-1	VioBlue	REA810	Miltenyi Biotec	1:500
CD11b	VioBlue	REA592	Miltenyi Biotec	1:500
TCR-β	VioBlue	REA318	Miltenyi Biotec	1:100
B220	VioBlue	REA755	Miltenyi Biotec	1:500
CD335	VioBlue	REA815	Miltenyi Biotec	1:50
Sca-1	PE-CF594	D7	BD Bioscience	1:40
Human HSC, CD45^{hi} cells				
CD45	FITC	REA747	Miltenyi Biotec	1:140
CD34	PE-Cy7	581	BioLegend®	1:20

Supplementary Table 3. Characteristic cell surface markers for the identification of mouse BM cells with flow cytometry. Abbreviations: long-term (LT) and short-term (ST) hematopoietic stem cell (HSC), multipotent progenitor (MPP), common myeloid progenitor (CMP), granulocyte monocyte progenitor (GMP).

Cell Type	Flow cytometry markers
Mouse	
MSC	Lin- CD105+ CD271+ CD90.2+
LT-HSC	Lin- Sca+ cKit+ CD150+ CD48-
ST-HSC	Lin- Sca+ cKit+ CD150- CD48-
MPP2	Lin- Sca+ cKit+ CD150+ CD48+
MPP3	Lin- Sca+ cKit+ CD150- CD48+
CMP / GMP	Lin- Sca- cKit+ CD34+
Monocytes	CD45+ CD11b+ Ly6C^{Hi} Ly6G- F4/80^{Lo}
Macrophages	CD45+ CD11b+ Ly6C^{Lo} Ly6G- F4/80+
Neutrophils	CD45+ CD11b+ Ly6C+ Ly6G+ F4/80-
Dendritic cells	CD11c+ CD11b- F4/80- MHC-II^{Hi}
Human	
hHSPC	SSC^{Lo} CD45^{Lo} CD34+
CD45^{hi} cells	CD45^{hi} CD34-

Supplementary Table 4. Overview of the used primers.

Name	Sequence	Tm (°C)*
Eef2_F	TGTCAGTCATCGCCCATGTG	57.6
Eef2_R	CATCCTTGCGAGTGTCAGTGA	57.1
SL-RNA_F	AACTAACGCTATATAAGTAT	42.6
SL-RNA_R	CAATAAAGTACAGAAACTG	43.1

*Melting temperatures of primers.
