# Supplementary material for: Long-term hematopoietic stem cells as a parasite niche during treatment failure in visceral leishmaniasis
Source: Commun Biol. 2022 Jun 25;5:626. doi: 10.1038/s42003-022-03591-7 (PMC9233693; doi:10.1038/s42003-022-03591-7)
Supplement: Supplementary file 2 — Description of Additional Supplementary Files [file 42003_2022_3591_MOESM2_ESM.pdf]

Description of Additional Supplementary Files

File name: Supplementary Data 1
Description: List of compounds and their dose tested in Syrian golden hamsters. Percentage of recovery (%R), single injection dose (SID), twice daily injection dose (BID).

File name: Supplementary Data 2
Description: Differential LT-HSC genes and systems biology analyses to evaluate enrichment of hallmark gene sets, pathways, transcription factor motifs, cell type enrichment and cross-pathology overlap.

File name: Supplementary Data 3
Description: The source data of Figures 1-6 and Supplementary Figures 1-6.
