# Supplementary material for: Long-term hematopoietic stem cells as a parasite niche during treatment failure in visceral leishmaniasis
Source: Commun Biol. 2022 Jun 25;5:626. doi: 10.1038/s42003-022-03591-7 (PMC9233693; doi:10.1038/s42003-022-03591-7)
Supplement: Supplementary file 6 — Reporting Summary [file 42003_2022_3591_MOESM6_ESM.pdf]

nature research

Corresponding author(s): Guy Caljon
Last updated by author(s): May 4, 2022

Reporting Summary

Nature Research wishes to improve the reproducibility of the work that we publish. This form provides structure for consistency and transparency in reporting. For further information on Nature Research policies, see our Editorial Policies and the Editorial Policy Checklist.

Statistics

For all statistical analyses, confirm that the following items are present in the figure legend, table legend, main text, or Methods section.

n/a	Confirmed	
☐	☒	The exact sample size (n) for each experimental group/condition, given as a discrete number and unit of measurement
☐	☒	A statement on whether measurements were taken from distinct samples or whether the same sample was measured repeatedly
☐	☒	The statistical test(s) used AND whether they are one- or two-sided<br>*Only common tests should be described solely by name; describe more complex techniques in the Methods section.*
☒	☐	A description of all covariates tested
☒	☐	A description of any assumptions or corrections, such as tests of normality and adjustment for multiple comparisons
☐	☒	A full description of the statistical parameters including central tendency (e.g. means) or other basic estimates (e.g. regression coefficient) AND variation (e.g. standard deviation) or associated estimates of uncertainty (e.g. confidence intervals)
☒	☐	For null hypothesis testing, the test statistic (e.g. F, t, r) with confidence intervals, effect sizes, degrees of freedom and P value noted<br>*Give P values as exact values whenever suitable.*
☒	☐	For Bayesian analysis, information on the choice of priors and Markov chain Monte Carlo settings
☒	☐	For hierarchical and complex designs, identification of the appropriate level for tests and full reporting of outcomes
☒	☐	Estimates of effect sizes (e.g. Cohen's d, Pearson's r), indicating how they were calculated

Our web collection on statistics for biologists contains articles on many of the points above.

Software and code

Policy information about availability of computer code

Data collection: *Provide a description of all commercial, open source and custom code used to collect the data in this study, specifying the version used OR state that no software was used.*

Data analysis: *Provide a description of all commercial, open source and custom code used to analyse the data in this study, specifying the version used OR state that no software was used.*

For manuscripts utilizing custom algorithms or software that are central to the research but not yet described in published literature, software must be made available to editors and reviewers. We strongly encourage code deposition in a community repository (e.g. GitHub). See the Nature Research guidelines for submitting code & software for further information.

Data

Policy information about availability of data

All manuscripts must include a data availability statement. This statement should provide the following information, where applicable:

- Accession codes, unique identifiers, or web links for publicly available datasets
- A list of figures that have associated raw data
- A description of any restrictions on data availability

The authors declare that the data underlining the findings of this study are available within the paper and its supplementary information files. The source data underline Figures 1-6 and Supplementary Figures 1-6 are provided as source data file.

Field-specific reporting

Please select the one below that is the best fit for your research. If you are not sure, read the appropriate sections before making your selection.

☒ Life sciences ☐ Behavioural & social sciences ☐ Ecological, evolutionary & environmental sciences

For a reference copy of the document with all sections, see nature.com/documents/nr-reporting-summary-flat.pdf

Life sciences study design

All studies must disclose on these points even when the disclosure is negative.

Sample size	All vitro experiments and analyses have been performed between 2-6 times, each time as single or duplicate depending of the experimental conditions. For vivo work 3-6 animals in each group have been used.
Data exclusions	No data was excluded from any analysis.
Replication	All experiments have been repeated 2-6 times as mentioned in the sample size section above.
Randomization	Animals were allocated in experimental groups based on simple randomization.
Blinding	Blinding was not relevant to the study as no experimental vivo groups were compared.

Reporting for specific materials, systems and methods

We require information from authors about some types of materials, experimental systems and methods used in many studies. Here, indicate whether each material, system or method listed is relevant to your study. If you are not sure if a list item applies to your research, read the appropriate section before selecting a response.

Materials & experimental systems

n/a	Involved in the study	
☐	☒	Antibodies
☐	☒	Eukaryotic cell lines
☒	☐	Palaeontology and archaeology
☐	☒	Animals and other organisms
☐	☒	Human research participants
☒	☐	Clinical data
☒	☐	Dual use research of concern

Methods

n/a	Involved in the study	
☒	☐	ChIP-seq
☐	☒	Flow cytometry
☒	☐	MRI-based neuroimaging

Antibodies

Antibodies used	Specific antibodies against: cKit (PE-Vio770, REA791, Miltenyi Biotec), CD150 (APC, REA299, Miltenyi Biotec), CD48 (PerCP-Vio700, HM48-1, Miltenyi Biotec) CD34 (FITC, REA383, Miltenyi Biotec), Ter119 (VioBlue, Ter-119, Miltenyi Biotec), Gr-1 (VioBlue, REA810, Miltenyi Biotec), CD11b (VioBlue, REA592, Miltenyi Biotec), TCR-β (VioBlue, REA318, Miltenyi Biotec), B220 (VioBlue, REA755, Miltenyi Biotec), CD335 (VioBlue, REA815, Miltenyi Biotec), Sca1 (APC-Cy7, D7, BD Biosciences), Ly6C (APC, AL-21, BD Biosciences), Ly6G (APC-Vio770, REA526, Miltenyi Biotec), CD11b (PerCP, M1/70, BioLegend®), F4/80 (PE-Cy7, BM8, BioLegend®), CD45 (VioGreen, REA737, Miltenyi Biotec), CD105 (PE-Vio770, REA1058, Miltenyi Biotec), CD271 (APC-Vio770, REA648, Miltenyi Biotec), CD11c (APC-Cy7, REA791, Miltenyi Biotec), MHC-II (APC, M5/114.15.2, Miltenyi Biotec)
Validation	All antibodies obtained from various companies have been validated in the past by them or before the start of experiments in this paper.

Eukaryotic cell lines

Policy information about cell lines

Cell line source(s)	The L. infantum strain MHOM/FR/96/LEM3323, with an inherent Sb resistance, was obtained from a HIV-positive patient from the Languedoc area in Southern France and kindly provided by CNRL (Montpellier, France). The L. donovani strain MHOM/ET/67/L82 was isolated from an Ethiopian VL-patient.
Authentication	All strains were authentified by qPCR in past studies.
Mycoplasma contamination	Parasite cultures were free of Mycoplasma.

Commonly misidentified lines
(See ICLAC register)

Name any commonly misidentified cell lines used in the study and provide a rationale for their use.

Animals and other organisms

Policy information about studies involving animals; ARRIVE guidelines recommended for reporting animal research

Laboratory animals

BALB/c mice were used, all female between 6-8 weeks at the start of infection.

Wild animals

Provide details on animals observed in or captured in the field; report species, sex and age where possible. Describe how animals were caught and transported and what happened to captive animals after the study (if killed, explain why and describe method; if released, say where and when) OR state that the study did not involve wild animals.

Field-collected samples

For laboratory work with field-collected samples, describe all relevant parameters such as housing, maintenance, temperature, photoperiod and end-of-experiment protocol OR state that the study did not involve samples collected from the field.

Ethics oversight

Ethical Committee (ECD) of the University of Antwerp.

Note that full information on the approval of the study protocol must also be provided in the manuscript.

Human research participants

Policy information about studies involving human research participants

Population characteristics

Bone marrow of patients with suspected mastocytosis was used, residual samples were delivered 'blind', with no information regarding age or gender.

Recruitment

Healthy subjects were included in this project. Residual bone marrow aspirates were used that were collected for diagnostic purposes from patients with suspected mastocytosis.

Ethics oversight

Bone marrow aspirate rest samples were available for in vitro infection experiments following approval by the Committee of Medical Ethics UZA-UA (B3002021000027).

Note that full information on the approval of the study protocol must also be provided in the manuscript.

Flow Cytometry

Plots

Confirm that:

☒ The axis labels state the marker and fluorochrome used (e.g. CD4-FITC).

☒ The axis scales are clearly visible. Include numbers along axes only for bottom left plot of group (a 'group' is an analysis of identical markers).

☒ All plots are contour plots with outliers or pseudocolor plots.

☒ A numerical value for number of cells or percentage (with statistics) is provided.

Methodology

Sample preparation

Cell suspensions , i.e. bone marrow (either lineage depleted or derived macrophages and dendritic cells) in 2×107/mL concentration were treated with FcɣR-blocking agent (anti-CD16/32, clone 2.4G2, BD Biosciences) for 15 min, followed by a washing step using 500×g centrifugation and resuspension in PBS + 0.2% BSA buffer. Next, cells were incubated for 20 min at 4°C with a mix of fluorescent conjugated anti-mouse antibodies (Supplementary Table S2) at optimized concentrations. DAPI Staining Solution (Miltenyi Biotec) was used to assess viability.

Instrument

MACSQuant Analyzer 10 (Miltenyi Biotec) serienummer: 21316, and FACSMelody (BD Bioscience) serienummer: R6627540032

Software

FlowLogic Software (Miltenyi Biotec)

Cell population abundance

Sorting was performed following the 'purity' protocol in the FACSMelody software. The quality of sorting was confirmed by analyzing post-sort samples.

Gating strategy

Gating strategy is provided in the supplementary information, briefly BM cells were separated into lineage positive (Lin+) and negative (Lin-) cells using negative magnetic sorting. For both fractions, a cell gate was selected in a SSC-A versus FSC-A plot, followed by a gating on singlets in a FSC-H versus FSC-A plot. Lin+ live (DAPI-) CD45+ cells were further characterized as neutrophils (CD11b+ Ly-6G+), monocytes (Ly-6Chi) and macrophages (SSClow, Ly-6C-, F4/80+). Lin- live (DAPI-) cells were either plotted as cKit versus Sca1 or as CD105 versus CD271 to allow identification of hematopoietic stem cells or mesenchymal stem cells, respectively. cKit+Sca1+ cells were further characterized as LT-HSCs (CD48- CD150+), ST-HSCs (CD48- CD150-), MPP2 (CD48+ CD150+), and MPP3 (CD48+ CD150-). cKit+Sca1- cells were identified as CMP/GMP. CD105 +CD271+ cells were further characterized as MSCs (CD90.2+). Next, BM cells were differentiated into dendritic cells or macrophages using GM-CSF or L929, respectively. For both subsets, a cell gate was selected in a SSC-A versus FSC-A plot,

followed by gating on singlets in a FSC-H versus FSC-A plot and a live (DAPI-) selection as in (A). CD45+ cells were further characterized as macrophages (CD11b+ F4/80+), and CD11c+ cells as dendritic cells (CD11b- F4/80- MHC-IIhi). Human BM aspirate was treated twice with erythrocyte lysis buffer, a SSC low cell gate was selected in a SSC-A versus FSC-A plot, followed by gating on singlets in a FSC-H versus FSC-A plot. Live (DAPI-) cells were further characterized as HSCs (CD45lo CD34+) and CD45hi cells (CD45hi CD34-).

☒ Tick this box to confirm that a figure exemplifying the gating strategy is provided in the Supplementary Information.
